# Utilization of circulating cell-free DNA profiling to guide first-line chemotherapy in advanced lung squamous cell carcinoma

**DOI:** 10.7150/thno.51243

**Published:** 2021-01-01

**Authors:** Tao Jiang, Liyan Jiang, Xiaorong Dong, Kangsheng Gu, Yueyin Pan, Qin Shi, Guojun Zhang, Huijuan Wang, Xiaochun Zhang, Nong Yang, Yuping Li, Jianping Xiong, Tienan Yi, Min Peng, Yong Song, Yun Fan, Jiuwei Cui, Gongyan Chen, Wei Tan, Aimin Zang, Qisen Guo, Guangqiang Zhao, Ziping Wang, Jianxing He, Wenxiu Yao, Xiaohong Wu, Kai Chen, Xiaohua Hu, Chunhong Hu, Lu Yue, Da Jiang, Guangfa Wang, Junfeng Liu, Guohua Yu, Junling Li, Henghui Zhang, Lihong Wu, Lu Fang, Dandan Liang, Yi Zhao, Weihong Zhao, Wenmin Xie, Shengxiang Ren, Caicun Zhou

**Affiliations:** 1 Department of Medical Oncology, Shanghai Pulmonary Hospital, Thoracic Cancer Institute, Tongji University School of Medicine, Shanghai, China.; 2 Department of Respiration, Shanghai Chest Hospital, Shanghai, China.; 3 Cancer Center, Union Hospital of Tongji Medical College, Huazhong University of Science and Technology, Wuhan, Hubei, China.; 4 Department of Medical Oncology, The First Affiliated Hospital of Anhui Medical University, Hefei, Anhui, China.; 5 Department of Chemotherapy, Anhui Provincial Hospital, Hefei, Anhui, China.; 6 Department of Oncology, Fuzhou Pulmonary Hospital of Fujian, Fuzhou, Fujian, China.; 7 Department of Respiration, The First Affiliated Hospital of Zhengzhou University, Zhengzhou, Henan, China.; 8 Department of Respiration, Henan Cancer Hospital, Zhengzhou, Henan, China.; 9 Medical Oncology, The Affiliated Hospital of Qingdao University, Qingdao, Shandong, China.; 10 Department of Medical Oncology, Hunan Cancer Hospital, Changsha, China.; 11 Department of Pulmonary and Critical Care Medicine, The First Affiliated Hospital of Wenzhou Medical College, Wenzhou, Zhejiang, China.; 12 Department of Medical Oncology, The First Affiliated Hospital of Nanchang University, Nanchang, Jiangxi, China.; 13 Department of Oncology, Xiang Yang Central Hospital, Xiangyang, Hubei, China.; 14 Department of Oncology, Renmin Hospital of Wuhan University, Wuhan, Hubei, China.; 15 Department of Respiration, General Hospital of Eastern Theater Command of Chinese People's Liberation Army, Nanjing, Jiangsu, China.; 16 Department of Medical Oncology, Zhejiang Cancer Hospital, Hangzhou, China.; 17 Cancer Center, The First Bethune Hospital of Jilin University, Changchun, Jilin, China.; 18 Department of Oncology, Harbin Medical University Cancer Hospital, Harbin, China.; 19 Department of Respiratory Medicine, Weifang People's Hospital, Weifang, Shandong, China.; 20 Medical Oncology, Affiliated Hospital of Hebei University, Baoding, Hebei, China.; 21 Department of Internal Medicine, Shandong Cancer Hospital & Institute, Jinan, Shandong, China.; 22 Department of Thoracic Surgery, The Third Affiliated Hospital of Kunming Medical University, Kunming, Yunnan, China.; 23 Medical Oncology, Beijing Cancer Hospital, Beijing, China.; 24 Department of Thoracic Surgery and Oncology, The First Affiliated Hospital of Guangzhou Medical University, Guangzhou, Guangdong, China.; 25 Department of Chemotherapy, Sichuan Cancer Hospital & Institute, Chengdu, Sichuan, China.; 26 Department of Oncology, The Fourth People's Hospital of Wuxi, Wuxi, Jiangsu, China.; 27 Medical Oncology, The First Affiliated Hospital of Soochow University, Suzhou, Jiangsu, China.; 28 Department of Medical Oncology, The First Affiliated Hospital of Guangxi Medical University, Nanning, Guangxi, China.; 29 Department of Oncology, The Second Xiangya Hospital of Central South University, Changsha, Hunan, China.; 30 Department of Oncology, Qingdao Municipal Hospital, Qingdao, Shandong, China.; 31 Medical Oncology, The Fourth Hospital of Hebei Medical University, Shijiazhuang, Hebei, China.; 32 Department of Respiration, Peking University First Hospital, Beijing, China.; 33 Department of Thoracic Surgery, 4th Hospital of Hebei Medical University, Shijiazhuang, Hebei, China.; 34 Department of Oncology, Weifang People's Hospital, Weifang, Shandong, China.; 35 Medical Oncology, Cancer Hospital Chinese Academy of Medical Sciences, Beijing, China.; 36 Beijing Genecast Biotechnology Co., Beijing, China.; 37 Nanjing Luye Pharmaceutical Co. Ltd, Nanjing, Jiangsu, China.

**Keywords:** Non-small-cell lung cancer, cell-free DNA, chemotherapy, machine learning

## Abstract

**Rationale:** Platinum-based chemotherapy is one of treatment mainstay for patients with advanced lung squamous cell carcinoma (LUSC) but it is still a “one-size fits all” approach. Here, we aimed to investigate the predictive and monitoring role of circulating cell-free DNA (cfDNA) profiling for the outcome of first-line chemotherapy in patients with advanced LUSC.

**Methods:** Peripheral blood samples of 155 patients from a phase IV trial and 42 cases from an external real-world cohort were prospectively collected. We generated a copy number variations-based classifier via machine learning algorithm to integrate molecular profiling of cfDNA, named RESPONSE SCORE (RS) to predict the treatment outcome. To monitor the treatment efficacy, cfDNA samples collected at different time points were subjected to an ultra-deep sequencing platform.

**Results:** The results showed that patients with high RS showed substantially higher objective response rate than those with low RS in training set (*P* < 0.001), validation set (*P* < 0.001) and real-world cohort (*P* = 0.019). Furthermore, a significant difference was observed in both progression-free survival (training set, *P* < 0.001; validation set: *P* < 0.001; real-world cohort: *P* = 0.019) and overall survival (training set, *P* < 0.001; validation set: *P* = 0.037) between high and low RS group. Notably, variant allele frequency (VAF) calculated from an ultra-deep sequencing platform significantly reduced in patients experienced a complete or partial response after 2 cycles of chemotherapy (*P* < 0.001), while it significantly increased in these of non-responder (*P* < 0.001). Moreover, VAF undetectable after 2 cycles of chemotherapy was correlated with markedly better objective response rate (*P* < 0.001) and progression-free survival (*P* < 0.001) than those with detectable VAF.

**Conclusions:** These findings indicated that the RS, a circulating cfDNA sequencing-based stratification index, could help to guide first-line chemotherapy in advanced LUSC. The change of VAF is valuable to monitor the treatment response.

## Introduction

Lung squamous cell carcinoma (LUSC) is a common histological type of non-small-cell lung cancer (NSCLC) 1, 2. Unlike lung adenocarcinoma, most of LUSC does not harbor targetable driver mutations. Even though some of LUSC had driver mutations, targeted therapies are rarely used in this setting 3, 4. Recently, immunotherapy targeted programmed cell death 1 (PD-1) and its ligand (PD-L1) has shifted the treatment landscape in patients with advanced LUSC, but only ~20% of them got response to anti-PD-(L)1 monotherapy without biomarker selection 5-8. Therefore, platinum-based chemotherapy still plays an important role in the treatment for patients with LUSC as either front line or second ling setting 9. However, platinum-based chemotherapy in LUSC is still a “one-size fits all” approach. Although we endeavored to investigate single or combined molecular features to predict chemotherapy response, to date, none of them had been successfully implemented in clinical practice 9, 10. Recently, next generation sequencing (NGS) characterizes alterations in genome and demonstrated that tumor mutation burden (TMB) was associated with response to checkpoint inhibitors 11, 12. Furthermore, other genomic signature was found to be correlated with molecular targeted agents 13, which showed the potent for identifying efficacy predictors to chemotherapy via genetic profiling.

Circulating cell-free DNA (cfDNA) is a potential surrogate for the genomic profiling of tumor 14-17. Several publications have reviewed the clinical application of cfDNA in NSCLC, and suggested cfDNA as an alternative predictor for targeted therapy and immunotherapy 14, 18-20. Indeed, it is feasible for real-time monitoring of tumor relapse or disease progression 21-23. Moreover, our previous study in a limited number of NSCLC patients found that those with objective response to first-line chemotherapy have distinct mutational landscape of cfDNA when compared with non-responders 24, indicating cfDNA profiling might be a potential marker to guide chemotherapy in NSCLC.

To further investigate the predictive value of cfDNA profiling for doublet chemotherapy in patients with advanced LUSC, we conducted this biomarker exploratory analysis in patients from a randomized phase IV trial (named LIPUSU, NCT02996214). From the training cohort, we developed a copy number variations (CNV)-based classifier, named RESPONSE SCORE (RS, details are listed in the Methods: Definition and Algorithm of RESPONSE SCORE), via machine learning algorithm to integrate molecular profiling. Our results showed that patients with high RS showed significantly superior objective response rate (ORR), progression-free survival (PFS) and overall survival (OS) than those with low RS in training, validation set and an external real-world cohort. Notably, change of variant allele frequency (VAF) of common mutations could monitor response and might help to identify early disease progression of chemotherapy.

## Methods

### Patients' enrollment and sample collection

Eligible patients were consecutively enrolled from a randomized phase IV trial, named LIPUSU. The purpose of LIPUSU is to investigate the efficacy and safety of paclitaxel liposome injection plus cisplatin compared with gemcitabine plus cisplatin as first-line therapy in patients with advanced LUSC. Details of the study design and patient eligibility criteria were summarized in https://clinicaltrials.gov/ (NCT02996214). Briefly, enrolled patients were randomly assigned to receive up to 6 cycles of paclitaxel liposome (175 mg/m^2^) plus cisplatin at a dose of 75 mg/m^2^ (LP) on day 1, every 21 days, or gemcitabine 1000 mg/m^2^ (on day 1 and 8, every 21 days) plus cisplatin at a dose of 75 mg/m^2^ (GP), on day 1, every 21 days. Blood samples were collected at the baseline and two cycle of chemotherapy or disease progression. The study protocol was approved by the institutional ethics committee of each participating medical center. All patients signed informed consent forms before the initiation of any study-related procedure. We also adopted an external real-word cohort from online data to further validate the finding in this study.

### DNA extraction and sequencing

Peripheral blood cells and plasma were separated by centrifugation at 1600×g for 10 min. Supernatant plasma was transferred to a 2 milliliter (mL) centrifuge tube and centrifuged at 16,000×g for 10 min. MagMAXTM Cell-Free DNA isolation kit (Life Technologies, California, USA) was utilized to extract cfDNA in the plasma according to the instruction. TIANGEN whole blood DNA kit (TIANGEN, Beijing, China) was used to extract DNA from peripheral blood cells according to the manufacturer's instructions. Further details of sequencing and bioinformatic analyses were provided in Supplementary Methods.

### Definition and Algorithm of RESPONSE SCORE

To better predict the treatment response, we identified a set of genes to generate CNV-based classifier, named RESPONSE SCORE (RS). The criteria for the included genes were: (i) common driver mutations with frequency ≥ 2%; (ii) genes showed predictive value with *P* < 0.1 in univariate analysis from our cohort; (iii) genes potentially associated with efficacy of chemotherapy (e.g. ERCC1/2, BRAC1/2, RRM1, etc.) in previous studies; (iv) genes potentially correlated with the transport, metabolism and resistance of chemotherapeutic agents in previous publications; (v) genes involved in several biological processes associated with cancer cell survival, growth and apoptosis such as DNA replication, transcription and damage repair, cell cycle, immune response pathways and so on. The detailed algorithm of RS calculation was summarized in Supplemental Materials.

### Statistical analysis

Both Wilcoxon signed rank test and *t* test were applied for comparison of CNV and mutation frequency between defined patients' groups. Correlations between RS high and low group were analyzed using the chi-squared or Fisher's exact test for categorical variables. The continuous variables were analyzed by ANOVA and Tukey's multiple comparison tests. Mann-Whitney U tests or Kruskal-Wallis rank sum tests were used for comparisons of continuous variables across multiple groups. The Kaplan-Meier curve with log-rank test was used to test the significance of differences between two groups. All the diagrams were drawn with R packages including ComplexHeatmap and ClusterProfile. Circos-0.69-6 was used to generate circos plots for CNV distributions. All statistical analyses were conducted using GraphPad PRISM 6.0 and the SPSS statistical software, version 22.0 (SPSS Inc., Chicago, IL, USA). *P* < 0.05 was considered statistically significant.

## Results

### Baseline characteristics of included patients

**Figure 1:**
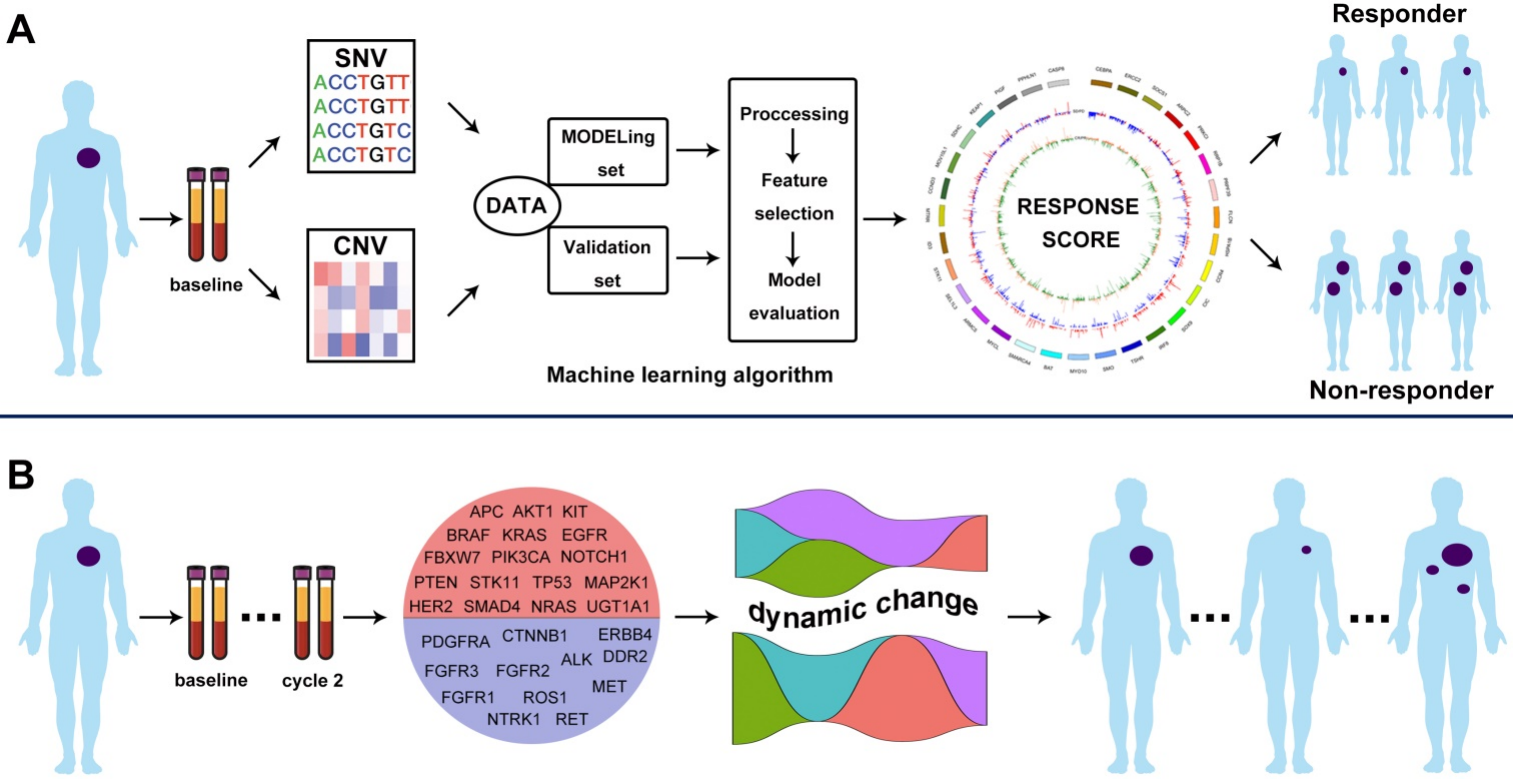
** Schematic illustration of the overall investigation. A.** Machine learning algorithm to generate CNV-based RS for response prediction via integrating cfDNA molecular features; **B.** ICP-based dynamic change of VAF as baseline and cycle 2 treatment monitored the treatment response.

Totally, 155 patients with advanced LUSC were identified and their blood samples at baseline and cycle 2 treatment were prospectively collected (Figure 1 and Figure S1). Baseline characteristics were summarized in Table S1. In brief, 151 (97.4%) of them were male and 129 (83.2%) had Eastern Cooperative Oncology Group (ECOG) performance status of 1. Most of patients had smoking history (96.1%). 80 patients received LP and 75 received GP. In LP group, there were 1, 47, 18 and 14 patients' that experienced complete response (CR), partial response (PR), stable disease (SD) and disease progression (PD) to first-line treatment, respectively. In GP group, 45, 10 and 20 patients experienced PR, SD and PD. Median PFS and OS were 153 and 341 days in LP group, 154 and 384 days in GP group, respectively.

### Mutational landscape of cfDNA and its association with treatment response

**Figure 2:**
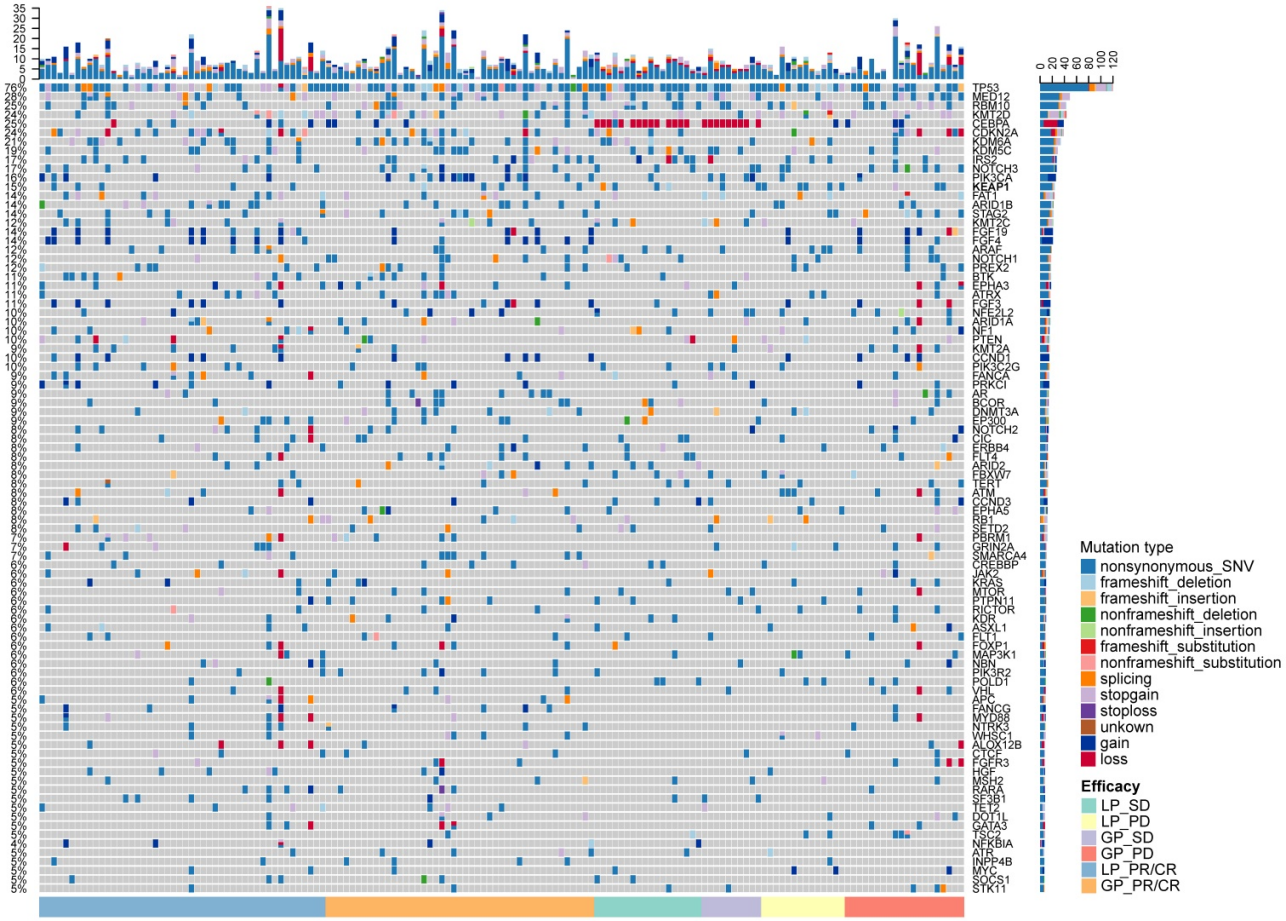
** The mutational landscape of included patients.** Upper panel: The frequency of listed driver genes. Middle panel: The matrix of mutations in a selection of frequently mutated genes. Columns represent samples. Right panel: The total number of patients harboring mutations in each gene. LP, paclitaxel liposome plus cisplatin; GP, gemcitabine plus cisplatin; CR, complete response; PR, partial response; SD, stable disease; PD, disease progression.

We identified 106 common genetic alterations with mutational frequency ≥ 2% (Figure 2). The most common genetic alteration was *TP53* (76.1%, 118/155). We listed the SNV and CNV landscape of LP and GP group in Figure S2-5. Overall, the mutational landscape was analogous between patients with CR/PR and SD/PD (Figure 2). The median TMB was 6.5 and 7.6 mutations/Mb in LP and GP group, respectively. While we used different cutoffs of TMB, it did not show any predictive value for both ORR and PFS (cutoff of TMB 25th, 50th and 75th: ORR, *P* = 0.793, *P* = 0.760,* P* = 0.880, respectively; PFS,* P* = 0.9267,* P* = 0.7128,* P* = 0.5887, respectively; Figure S6A-F). Subgroup analysis showed distinct cutoffs of TMB was also not associated with ORR and PFS in both LP (cutoff of TMB 25th, 50th and 75th: ORR, *P* = 0.598, *P* = 0.999,* P* = 0.598, respectively; PFS,* P* = 0.9701,* P* = 0.8431,* P* = 0.7685, respectively; Figure S7A-F) and GP group (cutoff of TMB 25th, 50th and 75th: ORR, *P* = 0.448, *P* = 0.925,* P* = 0.912, respectively; PFS,* P* = 0.6805,* P* = 0.5232,* P* = 0.8230, respectively; Figure S7G-L). Of note, when we investigated the predictive value of each prevalent gene alteration (frequency ≥ 5%), we found no individual gene alterations showed association with the outcome of chemotherapy.

### Generation of CNV-based RS for response prediction

**Figure 3:**
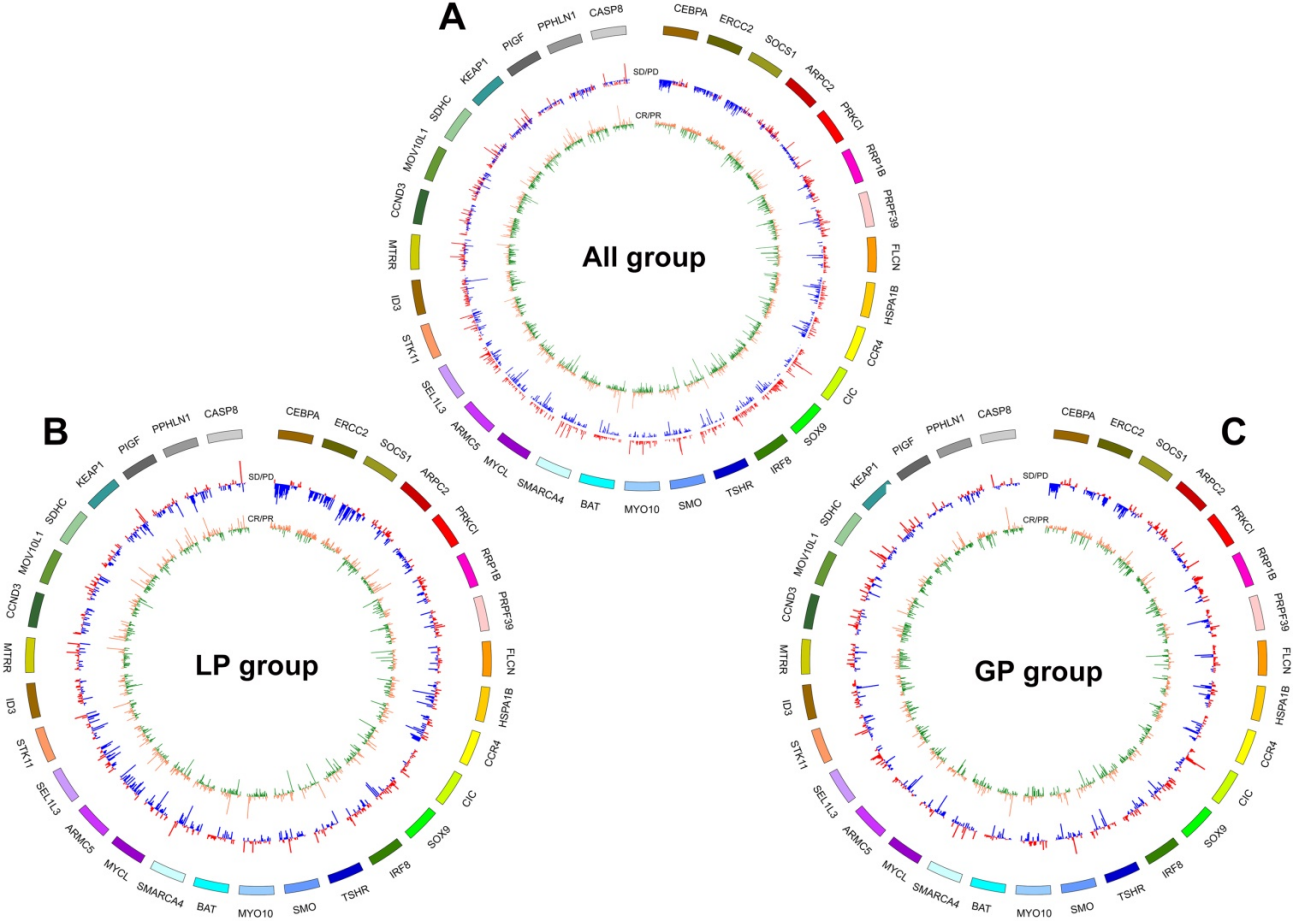
** CNV pattern of 31 genes including CASP8, PPHLN1, PIGF, KEAP1, SDHC, MOV10L1, CCND3, MTRR, ID3, STK11, SEL1L3, ARMC5, MYCL, SMARCA4, BAT, MYO10, SMO, TSHR, IRFB, SOX9, CIC, CCR4, HSPA1B, FLCN, PRPF39, RRP1B, PRKCI, ARPC2, SOCS1, ERCC2, CEBPA showed obviously distinct distribution between patients with CR/PR and SD/PD in all (A), LP (B) and GP (C) group.** From inside to out of each circus plot: the first circle represents the CNVs of patients in SD and PD group (orange represents amplification, green represents loss or deletion); the second circle represents the CNVs of patients in PR and CR group (red represents amplification, blue represents loss or deletion). Outermost circle represents the chromosomes. CR, complete response; PR, partial response; SD, stable disease; PD, disease progression. LP, paclitaxel liposome plus cisplatin; GP, gemcitabine plus cisplatin.

**Figure 4:**
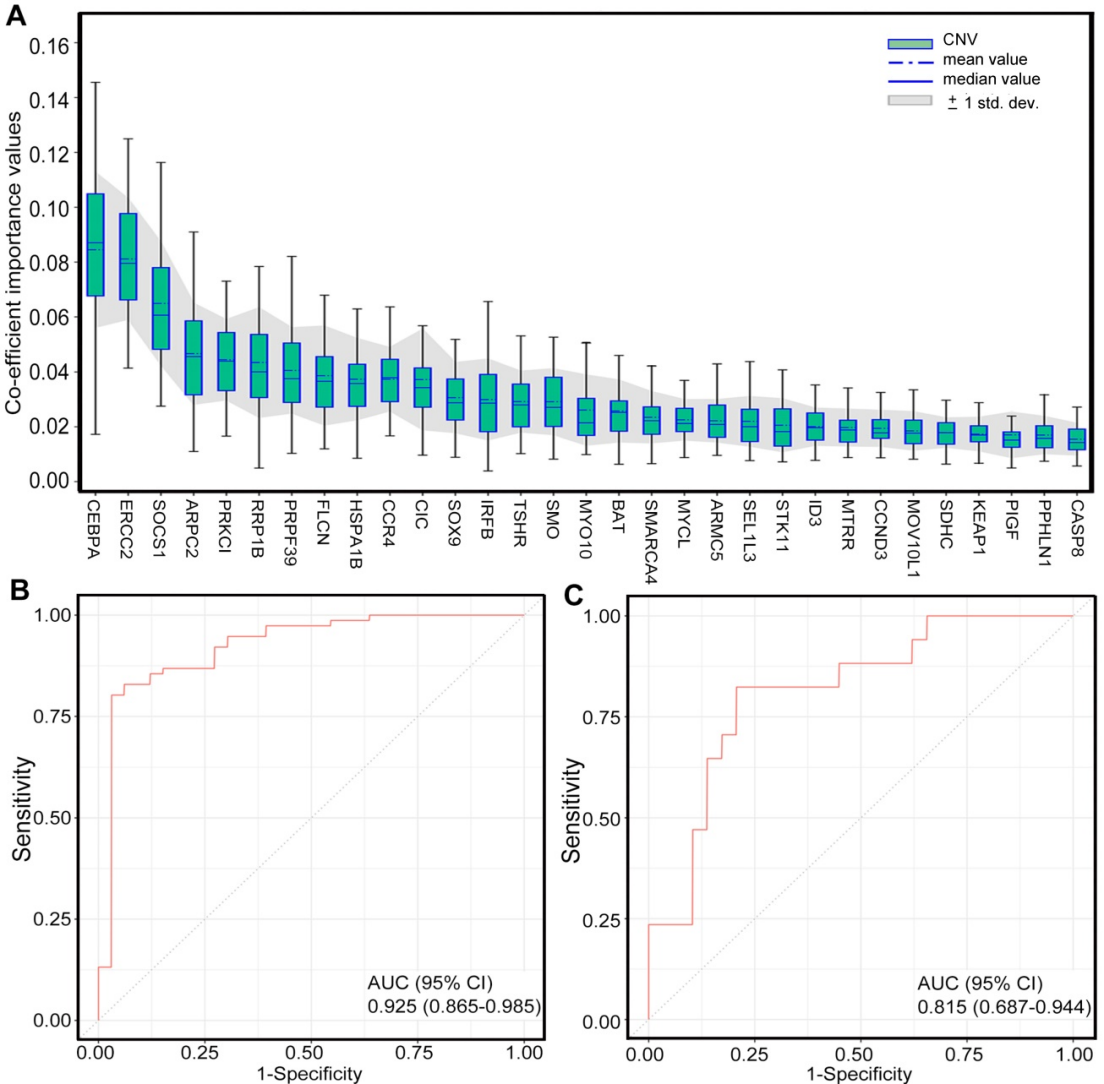
** Generation of CNV-based RS for response prediction**. **A.** Different co-efficient importance values in this model via selecting features with the best accuracy score in the ensemble or LASSO supervised method; **B.** Receiver operator characteristic curve analysis result in training set; **C.** Receiver operator characteristic curve analysis result in validation set. CNV, copy number variation; AUC, area under the ROC curve. Feature selection (Fig. A) was carried out with two steps. First, several statistical methods were utilized to evaluate the difference between two groups of samples in training set for each feature, including deviation, mutual information, AUC and p-values of Chi-Square test, Wilcoxon rank sum test, ANOVA and Student's t test, after which features with significantly different signal in at least four of criteria mentioned above were selected. Then, the method of LASSO was conducted to select features with the best accuracy score.

To identify the patients who might benefit from chemotherapy, we generated a CNV-based classifier, named RS (Figure 1A). Firstly, we focused on the potential impact of cfDNA concentrations. The results showed that baseline cfDNA concentrations had no significant difference between patients with CR/PR and SD/PD in all, LP and GP group (*P* > 0.05,* P* > 0.05,* P* > 0.05, respectively; Figure S8A-C). The median PFS was also similar among patients with different baseline cfDNA concentrations in three groups (*P* = 0.143,* P* = 0.656,* P* = 0.103, respectively; Figure S8D-F). Secondly, we found that the fraction of circulating tumor DNA (ctDNA) also had no significant difference between patients with CR/PR and SD/PD in all, LP and GP group (*P* > 0.05,* P* > 0.05,* P* > 0.05, respectively; Figure S9A-C). Moreover, the median PFS was also comparable among patients with different fraction of ctDNA in three groups (*P* = 0.975,* P* = 0.869,* P* = 0.834, respectively; Figure S9D-F). Finally, we excluded the potential impact of maximum VAF of SNV and CNV on therapeutic response (Figure S10). Following our above-mentioned defined criteria and strict algorithm, we identified CNV pattern of 31 genes including *CASP8, PPHLN1, PIGF, KEAP1, SDHC, MOV10L1, CCND3, MTRR, ID3, STK11, SEL1L3, ARMC5, MYCL, SMARCA4, BAT, MYO10, SMO, TSHR, IRFB, SOX9, CIC, CCR4, HSPA1B, FLCN, PRPF39, RRP1B, PRKCI, ARPC2, SOCS1, ERCC2 and CEBPA.* The results showed obviously different distribution between patients with CR/PR and SD/PD in all, LP and GP group (Figure 3A-C). These genes had different co-efficient importance values in this predictive model (Figure 4A) and the sum of co-efficient importance values based on the selected features for each sample was its individual RS. Receiver operator characteristic (ROC) curve analysis indicated that RS, the developed predictor in this study, could effectively distinguish patients with CR/PR from these with SD/PD in both training set [area under the ROC curve (AUC) = 0.925, Figure 4B] and validation set (AUC = 0.815, Figure 4C).

### Relationship between RS and treatment outcomes

**Figure 5:**
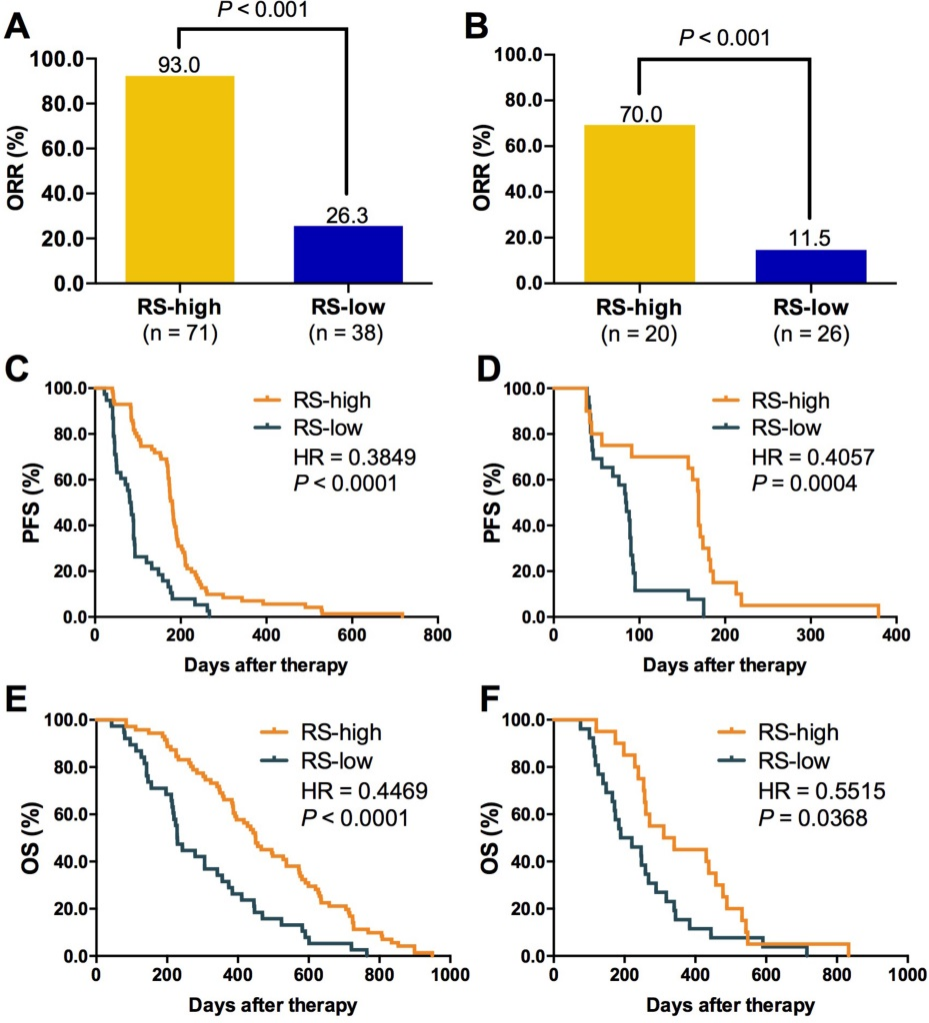
** Relationship between RS and treatment outcomes. A.** ORR comparison between RS high and low group in training set; **B.** ORR comparison between RS high and low group in validation set; **C.** Kaplan-Meier curve of PFS comparison between RS high and low group in training set; **D.** Kaplan-Meier curve of PFS comparison between RS high and low group in validation set; **E.** Kaplan-Meier curve of OS comparison between RS high and low group in training set; **F.** Kaplan-Meier curve of OS comparison between RS high and low group in validation set. RS, RESPONSE SCORE; ORR, objective response rate; PFS, progression-free survival; HR, hazard ratio. Unpaired student *t* test were applied for comparison of response rate between RS high and low groups. The Kaplan-Meier curve with log-rank test was used to test the significance of differences between two groups.

We then evaluated the relationship between RS and outcomes of first-line chemotherapy. The cutoff of RS was defined as the numerical value that showed the best accuracy and AUC in distinguishing patients with different treatment response. Therefore, all the patients in this study were divided into high or low RS group. As shown in Figure 5, patients with high RS showed markedly higher ORR than those with low RS in both training (93.0% *vs.* 26.3%, *P* < 0.001; Figure 5A) and validation set (70.0% *vs.* 11.5%, *P* < 0.001; Figure 5B). A significant difference was also observed in PFS (training set: HR = 0.38, *P* < 0.0001, Figure 5C; validation set: HR = 0.41, *P* = 0.0004, Figure 5D) and OS (training set: HR = 0.45, *P* < 0.0001, Figure 5E; validation set: HR = 0.55, *P* = 0.0368, Figure 5F) between two groups. Subgroup analysis in training set indicated that RS was a suitable predictor for both LP and GP group (Figure S11). However, RS could not distinguish the ORR (Figure S12A-B) and PFS (Figure S12C-D) of LP from GP in training and validation set.

### Validation of predictive value of RS in a real-word cohort

Considering the potential impact of different histology (*e.g.* LUSC *vs.* LUAD) and chemotherapeutic regimens, we further survey the universal significance of RS for predicting first-line chemotherapy outcomes in advanced NSCLC. We evaluated its predictive value in an external real-world cohort from previous publication^24^. 42 patients with advanced NSCLC received docetaxel plus cisplatin/carboplatin as first-line treatment (Figure S13A). The results showed that high RS was also correlated with significantly better ORR (54.5% *vs.* 15.0%, *P* = 0.019; Figure S13B) and PFS (HR = 0.42, *P* = 0.0023; Figure S13C) than those with low RS. These results suggested RS might be served as a universal predictor for first-line platinum-based doublet chemotherapy in advanced NSCLC and further investigation with large sample size is warranted.

### Change of VAF monitored the treatment response

**Figure 6:**
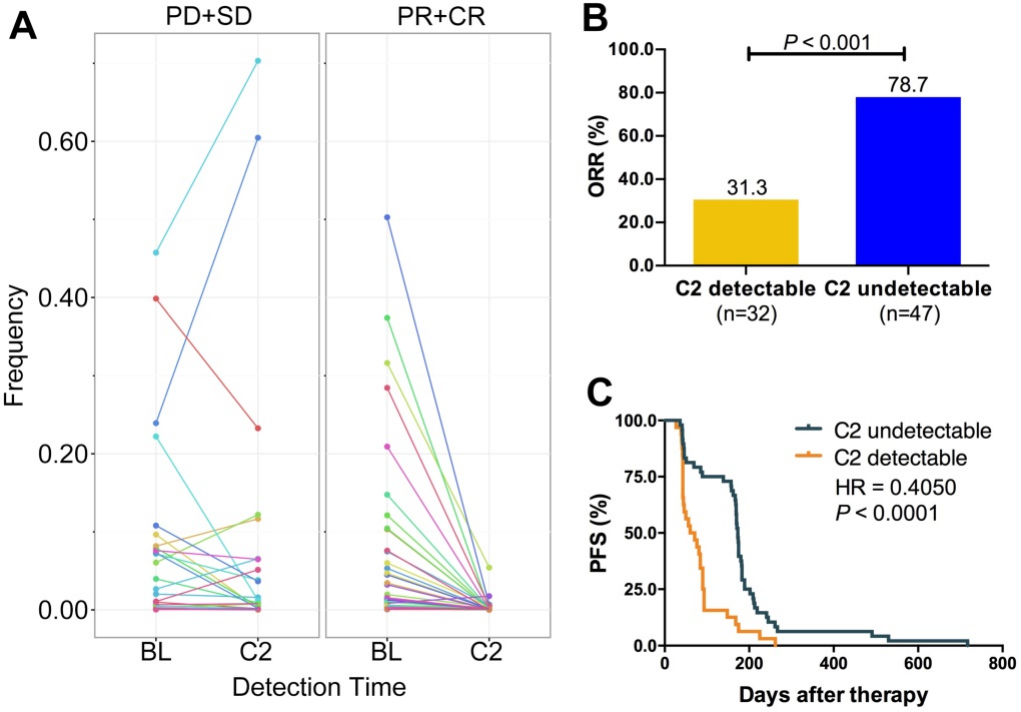
** ICP-based dynamic change of VAF monitored the treatment response. A.** ICP-based change of VAF between CR/PR and SD/PD at baseline and cycle 2 treatment. **B.** ORR comparison between VAF detectable and undetectable at cycle 2 treatment; **C.** Kaplan-Meier curve of PFS comparison between VAF detectable and undetectable at cycle 2 treatment. ORR, objective response rate; PFS, progression-free survival; BL, baseline; C2, cycle 2 treatment; CR, complete response; PR, partial response; SD, stable disease; PD, disease progression. Paired student *t* test were applied for the dynamic change of cfDNA VAF between baseline and C2 detection time. Unpaired student *t* test were applied for comparison of response rate between C2 detectable and undetectable groups. The Kaplan-Meier curve with log-rank test was used to test the significance of differences between two groups.

Several studies revealed that cfDNA dynamics could predict the treatment response of targeted therapies or immune checkpoint inhibitors. Here, we designed the Panel 2 covering 29 prevalent tumor related driver genes (Table S3) to explore whether changes of VAF in cfDNA could monitor chemotherapy response (Figure 1B). We collected eligible blood samples from 79 cases at baseline and cycle 2 treatment. Patients of responder (CR+PR) experienced a significant decrease of VAF while patients of non-responder (SD+PD) experienced an increase at cycle 2 treatment (Figure 6A). Moreover, patients of VAF undetectable at cycle 2 had significantly higher ORR (78.7%* vs.* 31.3%, *P* < 0.001; Figure 6B) and longer PFS (HR = 0.41, *P* < 0.0001; Figure 6C) than those of VAF detectable. Subgroup analysis showed that reduction of VAF was associated with durable clinical benefit in both LP (Figure S14A) and GP (Figure S14D) group. VAF undetectable at cycle 2 was correlated with substantially better ORR and PFS in both LP (ORR: 87.0% *vs.* 31.3%, *P* < 0.001, Figure S14B; PFS: HR = 0.36, *P* < 0.0001, Figure S14C) and GP group (ORR: 70.8% *vs.* 31.3%, *P* = 0.014, Figure S13E; PFS: HR = 0.46, *P* = 0.0089, Figure S14F) than those with detectable VAF. These findings suggested that changes of VAF in cfDNA could monitor the response to first-line chemotherapy in patients with advanced LUSC.

## Discussion

The current study comprehensively investigated the predictive value of cfDNA profiling for first-line platinum-based chemotherapy in patients with advanced LUSC. Our genetic analysis indicated that no single gene alternations were associated with outcome of chemotherapy and TMB could also not predict therapeutic response in patients with advanced LUSC. Alternatively, we generated a CNV-based classifier (RS) via machine learning algorithm to integrate cfDNA molecular profiling. We found that patients with high RS showed significantly superior ORR, PFS and OS than those with low RS in both training and validation set. We also validated these findings in an external real-world cohort. Notably, patients with objective response experienced a significant decrease of VAF after 2 cycles of treatment. Moreover, VAF undetectable at cycle 2 treatment was correlated with significantly better ORR and PFS than those with detectable VAF.

cfDNAs are derived from dying cells, detectable in plasma and are typically short DNA fragments (average length of 120-160 bp). In spite of the ambiguous biology of cfDNA, its clinical application (*e.g.* prediction or monitoring of treatment response, relapse, drug resistance, prognosis, etc.) has been extensively investigated 25. Using cfDNA as a predictor for therapeutic response in NSCLC has been investigated in many previous studies and majority of them focused on cfDNA levels 26, 27. However, a recent large-scale study found that the baseline cfDNA concentration did not validate its predictive value for outcome of systemic therapy in NSCLC 28. Meanwhile, dynamic changes in plasma cfDNA also did not correlate with radiologic response 28, suggesting that cfDNA concentration could not serve as a predictor of systemic therapy. Consistently, our results also found that the baseline cfDNA level cannot distinguish patients with CR/PR from those with SD/PD. Collectively, these results recommended that future studies on the predictive value of cfDNA should shift from its concentration or dynamics.

Single gene alterations as predictor for first-line chemotherapy in NSCLC went through a tortuous course and most of them finally failed 9. Our findings also showed that there were no frequent genetic mutations associated with the outcomes of chemotherapy in patients with advanced LUSC, suggesting that individual gene alterations showed very limited and inconsistent value for predicting outcomes of first-line chemotherapy in advanced LUSC. To improve the predictive power, we developed a CNV-based classifier (RS) via integrating cfDNA profiling in this study. CNV is considered as one of the major types of genome aberrations that contribute to tumorigenesis, maintenance and progression 29. Previous studies indicated that CNV pattern in cfDNA could act as a surrogate of primary tumor in various solid tumors 30, 31. Moreover, Louise *et al.* reported that CNV-based classification from circulating tumor cells could distinguish chemosensitive from chemorefractory cases with an accuracy of 83.3% in small cell lung cancer 32. In the current study, we integrated 31 frequent genes CNV as RS. As we mentioned above, most of them were involved in the carcinogenesis (*e.g. KEAP1, SMARCA4, MYCL, SOX9, STK11*), cell cycle regulation (*e.g. CCND3, SEL1L3*), DNA replication, transcription and damage repair (*e.g. PPHLN1, MOV10L1, ID3, CIC, FLCN, PRPF39, RRP1B, ARPC2, CEBPA*), immune response pathways (*e.g. IRF8, HSPA1B, CCR4, SOCS1*), chemotherapeutic drug transport and metabolism (*e.g. ERCC2, MTRR, PIGF, SDHC, TSHR*) in NSCLC. Although the association of each individual gene with treatment response was limited, a robust correlation between RS and chemotherapeutic response was observed. Patients with high RS had significantly better ORR, PFS and OS than those with low RS. Taken together, these findings indicated that this 31-genes CNV-based RS could be utilized to guide first-line chemotherapy in patients with advanced LUSC.

The genetic profiling of cfDNA also exhibited promising results for monitoring the efficacy of systemic therapy. Mok T *et al.* found that patients with circulating EGFR mutation clearance at cycle 3 had longer PFS and OS, suggesting that dynamic change of blood-based EGFR status could be a useful predictive marker 22. Several recent publications reported that plasma cfDNA profiling could also predict response to immune checkpoint inhibitors 23, 33-35. However, to date, no biomarkers have been developed to monitor the chemotherapy response in advanced LUSC. Our previous studies reported that the dynamic changes of *TP53* mutational burden might have monitoring value for the efficacy of first-line chemotherapy in advanced NSCLC. To improve its reliability, we optimized a small panel that included 29 frequent tumor related driver genes with an ultra-deep sequencing to increase the sensitivity of mutation detection. The result showed that change of VAF could effectively monitor the treatment response. Interestingly, Diehn *et al.* reported that cancer personalized profiling by deep sequencing (CAPP-seq) circulating tumor DNA (ctDNA) analysis could assess a response earlier than radiographic approaches and identify molecular residual disease after definitive therapy in patients with lung cancer 36, 37. These findings suggested that the application of customized panel of cfDNA sequencing could monitor the clinical benefits of chemotherapy.

There are several limitations that should be acknowledged. First, although the number of patients in the phase IV trial is large enough (n = 536), only 155 cases were included in this biomarker research, which may lead to the potential selection bias. However, when we compared the baseline features of two cohorts, we did not observe the obvious differences regarding to the baseline features including age, gender and ECOG PS. Second, we have utilized an external cohort to investigate the universal significance of RS for predicting first-line chemotherapy outcomes in advanced NSCLC regardless of histology. Due to the accessibility of sequencing data and clinical information for previous publication, only 42 cases were included, which is relatively small. A large prospective study is warranted in the future to validate the predictive efficacy of RS. Third, we only identified the CNV profile from cfDNA, whether it could well represent the CNV features in primary tumor cohort of LUSC remains future investigation. Last but not least, immunotherapy based combination therapy is the standard of care for advanced LUSC. Only to investigate the biomarkers to predict chemotherapy is less clinical significant nowadays in the era of immunotherapy. Nevertheless, platinum-based chemotherapy still plays an important role in the treatment for patients with LUSC considering the accessibility and price of immunotherapy in some areas of China. A substantial number of patients still need chemotherapy and are the potential population who benefit this biomarker analysis.

In summary, the current study indicated that cfDNA profiling is correlated with therapeutic response to first-line chemotherapy in patients with advanced LUSC. CNV-based RS showed potential value in predicting therapeutic effects, and change of VAF is valuable to monitor treatment response. These findings support the feasibility for utilization of cfDNA profiling to guide first-line chemotherapy in patients with advanced LUSC, and worth further validation in large scale population.

## Supplementary Material

Supplementary figures and tables.

## Abbreviations

cfDNA: cell-free DNA
CI: confidence interval
CNV: copy number variations
CR: complete response
DCR: disease control rate
ECOG PS: Eastern Cooperative Oncology Group performance status
HR: hazard ratios
ICP: individually customized panel
LUSC: lung squamous cell carcinoma
NGS: next generation sequencing
NSCLC: non-small-cell lung cancer
ORR: objective response rate
OS: overall survival
ROC: Receiver operator characteristic
RS: RESPONSE SCORE
PD: progressive disease
PD-1: programmed cell death 1
PD-L1: programmed cell death ligand 1
PR: partial response
SD: stable disease
TMB: tumor mutation burden
VAF: variant allele frequency
