# Supplementary material for: Utilization of circulating cell-free DNA profiling to guide first-line chemotherapy in advanced lung squamous cell carcinoma
Source: Theranostics. 2021 Jan 1;11(1):257–67. doi: 10.7150/thno.51243 (PMC7681090; doi:10.7150/thno.51243)
Supplement: Supplementary file 1 — Supplementary figures and tables. [file thnov11p0257s1.pdf]

SUPPLEMENTAL MATERIAL

Utilization of circulating cell-free DNA profiling to guide first-line chemotherapy in advanced lung squamous cell carcinoma

Supplemental methods..……………………………………………………………………….2
Supplemental Figure S1……………………………………………………………………….6
Supplemental Figure S2……………………………………………………………………….7
Supplemental Figure S3……………………………………………………………………….8
Supplemental Figure S4……………………………………………………………………….9
Supplemental Figure S5……………………………………………………………………...10
Supplemental Figure S6……………………………………………………………………...11
Supplemental Figure S7……………………………………………………………………...12
Supplemental Figure S8……………………………………………………………………...13
Supplemental Figure S9……………………………………………………………………...14
Supplemental Figure S10…………………………………………………………………….15
Supplemental Figure S11…………………………………………………………………….16
Supplemental Figure S12…………………………………………………………………….17
Supplemental Figure S13…………………………………………………………………….18
Supplemental Figure S14…………………………………………………………………….19
Supplemental Table S1……………………………………………………………………….21
Supplemental Table S2……………………………………………………………………….22
Supplemental Table S3……………………………………………………………………….24
References……………..…………………………………………………………………….25

Supplemental methods

DNA extraction and sequencing

Peripheral blood cells and plasma were separated by centrifugation at 1600×g for 10 min. Supernatant plasma was transferred to a 2 milliliter (mL) centrifuge tube and centrifuged at 16,000×g for 10 min. MagMAX™ Cell-Free DNA isolation kit (Life Technologies, California, USA) was utilized to extract cfDNA in the plasma according to the instruction. TIANGEN whole blood DNA kit (TIANGEN, Beijing, China) was used to extract genomic DNA from peripheral blood cells according to the manufacturer's instructions. DNA concentration was measured using Qubit dsDNA HS Assay kit or Qubit dsDNA BR Assay kit (Life Technologies, California, USA). Genomic DNA was sheared into 150-200 base pairs (bp) fragments with Covaris M220 Focused-ultrasonicator™ Instrument (Covaris, Massachusetts, USA). Fragmented genomic DNA and cfDNA libraries were constructed by KAPA HTP Library Preparation Kit (Illumina platforms) (KAPA Biosystems, Massachusetts, USA) following producer's instruction. DNA libraries from different samples were captured with two different panels. DNA libraries from baseline samples were captured with Panel 1, which was a 1.67 Mbp size panel covering exon regions of 543 genes (Supplemental Table S2; Genecast, Beijing, China) that included major tumor related genes, while DNA libraries from samples at baseline and the time after 2 cycles of chemotherapy were captured with Panel 2 (an ultra-deep sequencing platform), which was the ICP covering exon regions of 29 genes (Supplemental Table S3; Genecast, Beijing, China) that included prevalent tumor related driver genes. The captured samples were subjected to Illumina Novaseq 6000 for paired end sequencing.

Bioinformatics pipeline

For Panel 1, after filtering out low quality reads, clean paired-end reads generated from Novaseq platform were mapped to the hg19 reference genome with BWA 0.7.17 (default parameters), then Picard toolkit (version 2.1.0) was used for sorting, making duplicates. Genome Analysis ToolKit (version 3.7)(1) was used for realignment. VarDict (version 1.5.1)(2) was introduced for single nucleotide variation (SNV) calling while compound heterozygous mutations were merged with FreeBayes (version 1.2.0). Tumor-normal paired sample calling is processed during the mutation calling procedure, in order to filter out the personal germline mutations. The generated candidate mutations were annotated using ANNOVAR software tool(3), and then filtered by using the following criteria: A. germline mutation; B. support reads<5 or with strand bias; C. VAF < 0.5%; D. carrier ratio greater than 0.002 in the ExAC(4) and

gnomAD database. The resulted nonsynonymous mutations at the exonic regions were kept for TMB estimation.

We used an independently developed algorithm to determine CNV. Briefly, after correcting GC content and target region length, the read count for all target regions of each sample was normalized so that the different samples were comparable. Using the normalized read count, we constructed a baseline with 30 normal blood control samples. log2ratios between normalized test sample and control were calculated at each region-level first, and then merged to gene-level log2ratio. To determine the CNV for each gene, in addition to the absolute copy number, the gene specificity score (GCS) was calculated, with quantitating the instability of copy number and adding a statistical test filter to determine whether the GCS was significantly different from control samples. Only genes with statistical significance and the absolute copy number exceeding a given threshold would be judged to be CNV.

We also developed a novel method to estimate cancer cell fractions of cell-free DNA(5). A maximum likelihood model was built to estimate ctDNA fraction (CCF) based on informative SNPs, which were defined to be with significantly different variant allele frequency (VAF) in the paired blood cell and plasma samples. The hypothetic genotype of an informative SNP in cfDNA and ctDNA was determined by the VAF in the paired samples and the local copy number in the plasma sample. According to VAF, local copy number and hypothetic genotype, we clustered SNPs into multiple groups, representing different ctDNA sources, and calculated the likelihood of observing these SNPs under given CCFs in each cluster. CCF of each cluster could therefore be estimated by maximizing the likelihood. Cluster with the highest CCF was considered to be from the main source of ctDNA, and its CCF was then output as the final estimation.

For Panel 2, after filtering out low quality reads, clean paired-end reads generated from Novaseq platform were mapped to the hg19 reference genome with BWA 0.7.17. For genomic DNA, an adapted procedure based on Novosort (version 3.08) was used to remove duplications. For cfDNA, a series of Fgbio (Fulcrum Genomics) tools were used to group reads into families by Unique Molecular Identifier (UMI) and call consensus which was ready for variant calling. We used a customized variant calling pipeline for the present task, characterized by duplex UMI assisted deduplication, a set of customized filters and

matched genomic DNA. Variants in genomic DNA were called using VarScan (v2.4.2). For each candidate variant to be called in cfDNA, at least three consensus reads were required. Patient-matched genomic DNA and normal databases (1000 genome project, ExAC) (4) were utilized to remove common germline SNPs. After annotation with ANNOVAR, exonic and splicing variants were kept while variants with VAF > 15% and not in a pre-constructed white list consisting of 431 previously reported hotspot mutations(6, 7) were considered germline. Variants were marked and subjected to later filtering if falling in or partially overlapping a pre-defined blacklist which consists of repeat regions(8), segmental duplication and regions with low mappability(9). Then each yet surviving candidate variant was determined by a hypothesis test as whether significantly likely being sampled from a coordinate-specific background distribution built with 140 cfDNA healthy controls.

Definition and Algorithm of RESPONSE SCORE

In order to predict the therapeutic response of first-line chemotherapy, we randomly collected 109 samples (76 PR/CR, 33 SD/PD) to train random forest model. Another independent validation sample set including 46 samples (17 PR/CR, 29 SD/PD) was randomly collected to evaluate the performance of the constructed random forest model. Both of SNV and CNV information were used to construct the model. To shrink outliers and approximate Gaussian distribution, we calculated $\log_2(X+1)$ to represent the original value of each feature involved in model construction. We pre-processed the input data, first imputing missing data with median value of the corresponding feature and then standardizing values of each feature via subtracting the mean while dividing the standard deviation. Due to the sparse characteristics of SNV information, 272 SNV features were removed because only 2% samples had values of these SNV averagely. Feature selection was carried out with two steps. First, several statistic methods were utilized to evaluate the difference between two groups of samples in training set for each feature, including deviation, mutual information, AUC and p-values of Chi-Square test, Wilcoxon rank sum test, ANOVA and Student's t test, after which features with significantly different signal in at least four of criteria mentioned above were selected. Then, the method of LASSO was conducted to select features with the best accuracy score. After feature selection, 31 CNV and none of SNV features were retained to construct the model. We constructed random forest model with the strategy of leave-one-out cross-validation. In this procedure, hyper-parameter optimization was tuned via cross-validation grid exhaustive search with 40% of samples in test set. Independent samples were used as validation set to

evaluate performance of the constructed model based on the value of accuracy and the area under the curve (AUC). This analysis was implemented with Python 3.6, and this code is available at https://github.com/WellJoea/MLkit. The probability of having benefited therapeutic response of first-line chemotherapy predicted by the constructed model was defined as response score (RS) of each sample in this article.

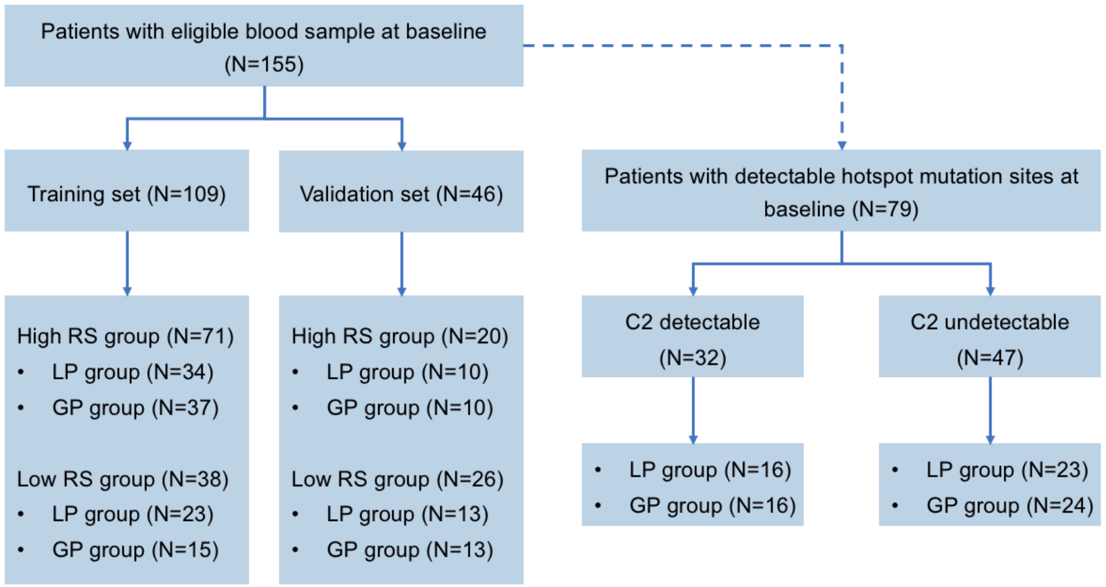

Supplemental Figure S1. Flowchart of patients' cohort. RS, RESPONSE SCORE; LP, paclitaxel liposome plus cisplatin; GP, gemcitabine plus cisplatin; C2, after 2 cycles treatment.

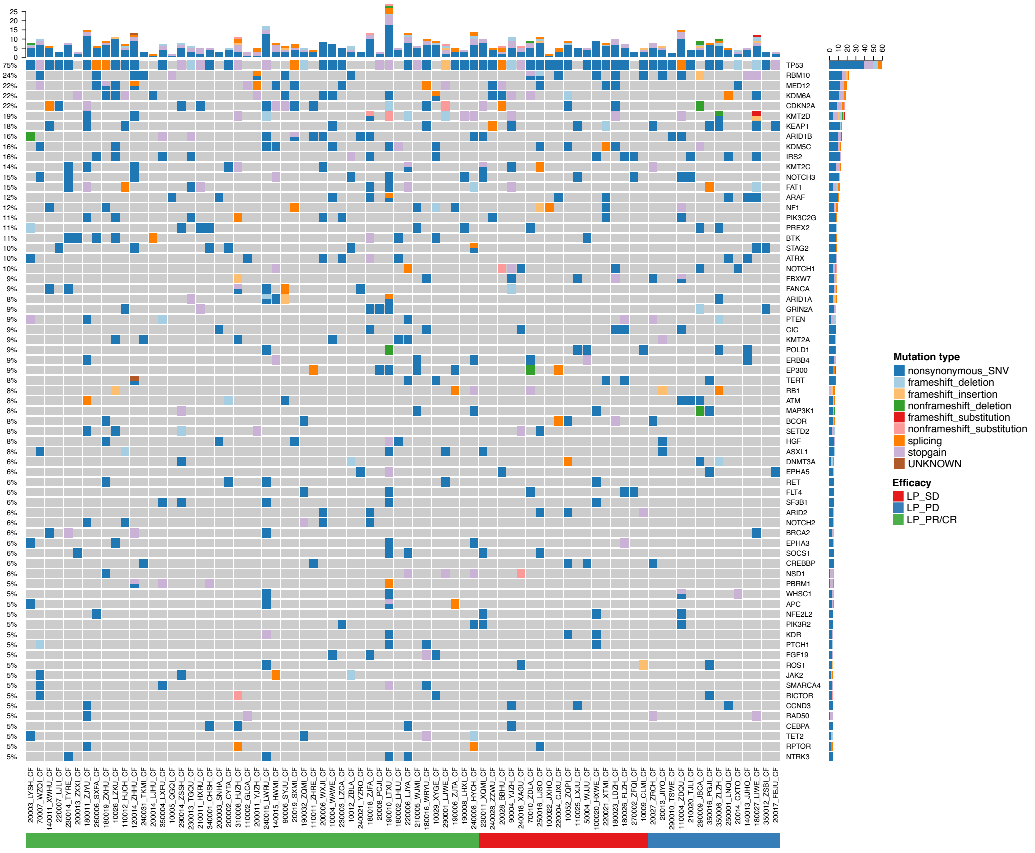

Supplemental Figure S2. SNV landscape of included patients in LP group. Upper panel: The frequency of listed driver genes. Middle panel: The matrix of mutations in a selection of frequently mutated genes. Columns represent samples. Right panel: The total number of patients harboring mutations in each gene. LP, paclitaxel liposome plus cisplatin; CR, complete response; PR, partial response; SD, stable disease; PD, disease progression.

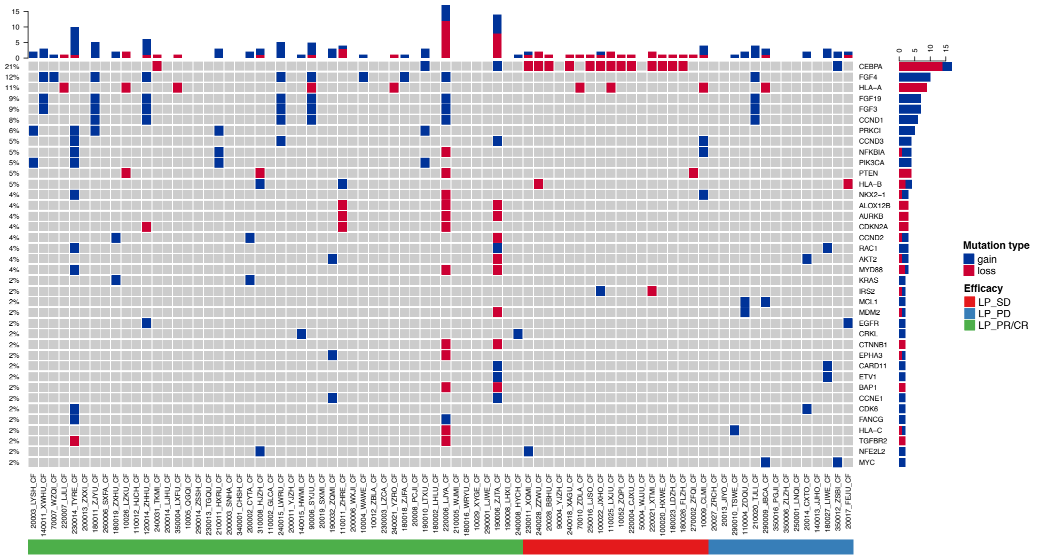

Supplemental Figure S3. CNV landscape of included patients in LP group. Upper panel: The frequency of listed driver genes. Middle panel: The matrix of mutations in a selection of frequently mutated genes. Columns represent samples. Right panel: The total number of patients harboring mutations in each gene. LP, paclitaxel liposome plus cisplatin; CR, complete response; PR, partial response; SD, stable disease; PD, disease progression.

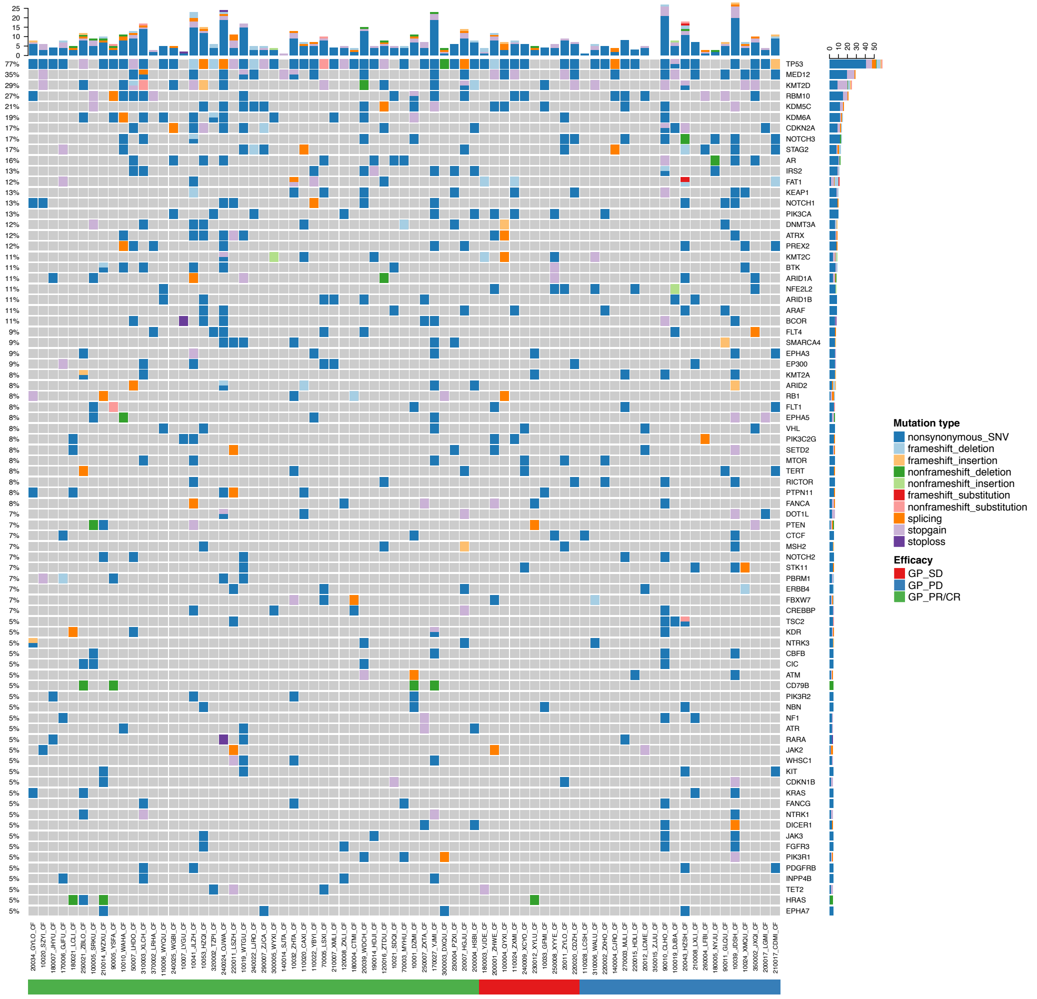

Supplemental Figure S4. SNV landscape of included patients in GP group. Upper panel: The frequency of listed driver genes. Middle panel: The matrix of mutations in a selection of frequently mutated genes. Columns represent samples. Right panel: The total number of patients harboring mutations in each gene. GP, gemcitabine plus cisplatin; CR, complete response; PR, partial response; SD, stable disease; PD, disease progression.

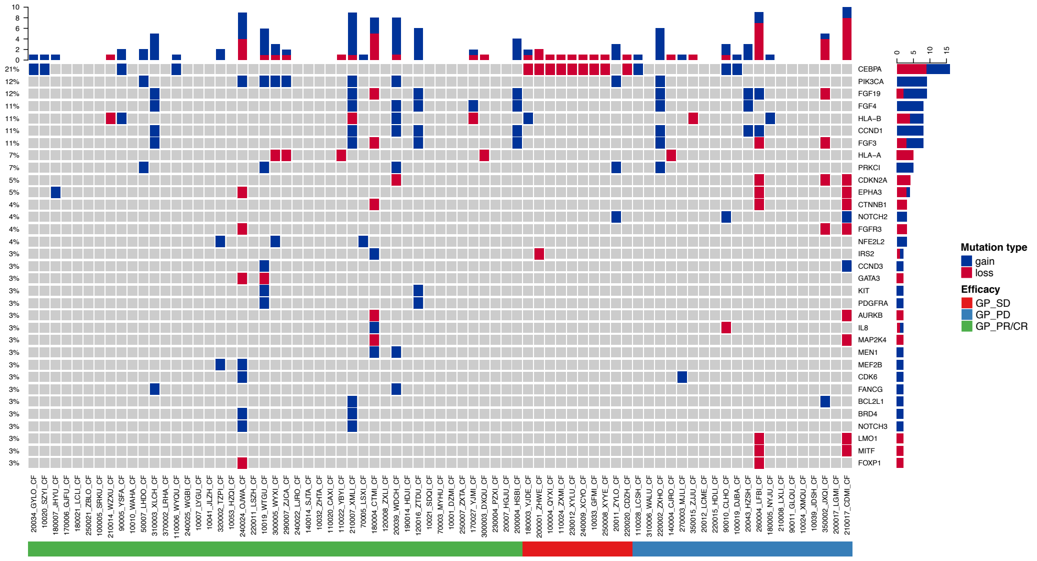

Supplemental Figure S5. CNV landscape of included patients in GP group. Upper panel: The frequency of listed driver genes. Middle panel: The matrix of mutations in a selection of frequently mutated genes. Columns represent samples. Right panel: The total number of patients harboring mutations in each gene. GP, gemcitabine plus cisplatin; CR, complete response; PR, partial response; SD, stable disease; PD, disease progression.

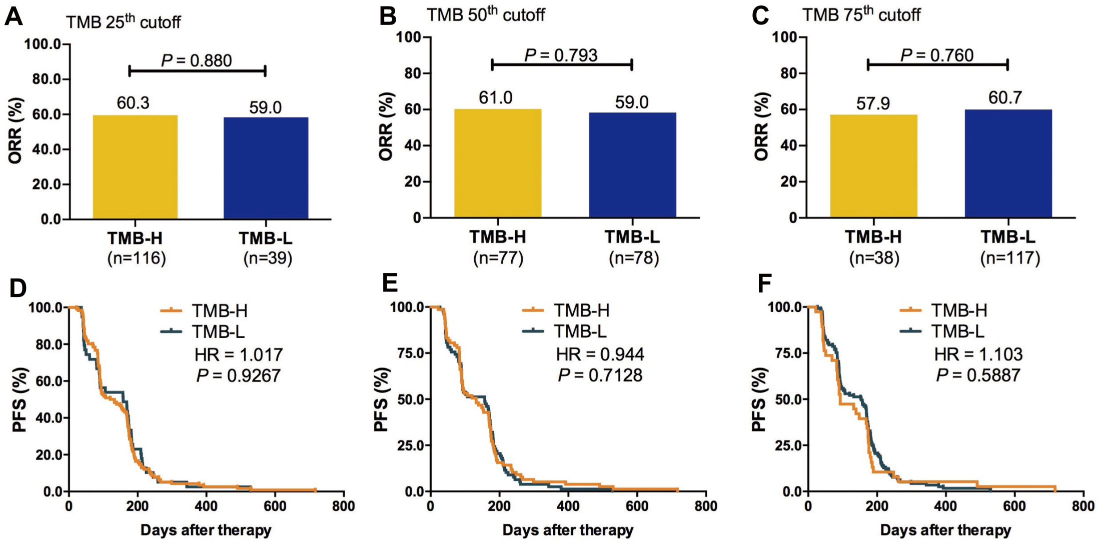

Supplemental Figure S6. Predictive value of TMB and single gene alterations. A-C. ORR comparison between TMB high and low group (A. TMB cutoff 25th percentile; B. TMB cutoff 50th percentile; C. TMB cutoff 75th percentile); D-F. PFS comparison between TMB high and low group (D. TMB cutoff 25th percentile; E. TMB cutoff 50th percentile; F. TMB cutoff 75th percentile). LP, paclitaxel liposome plus cisplatin; GP, gemcitabine plus cisplatin.

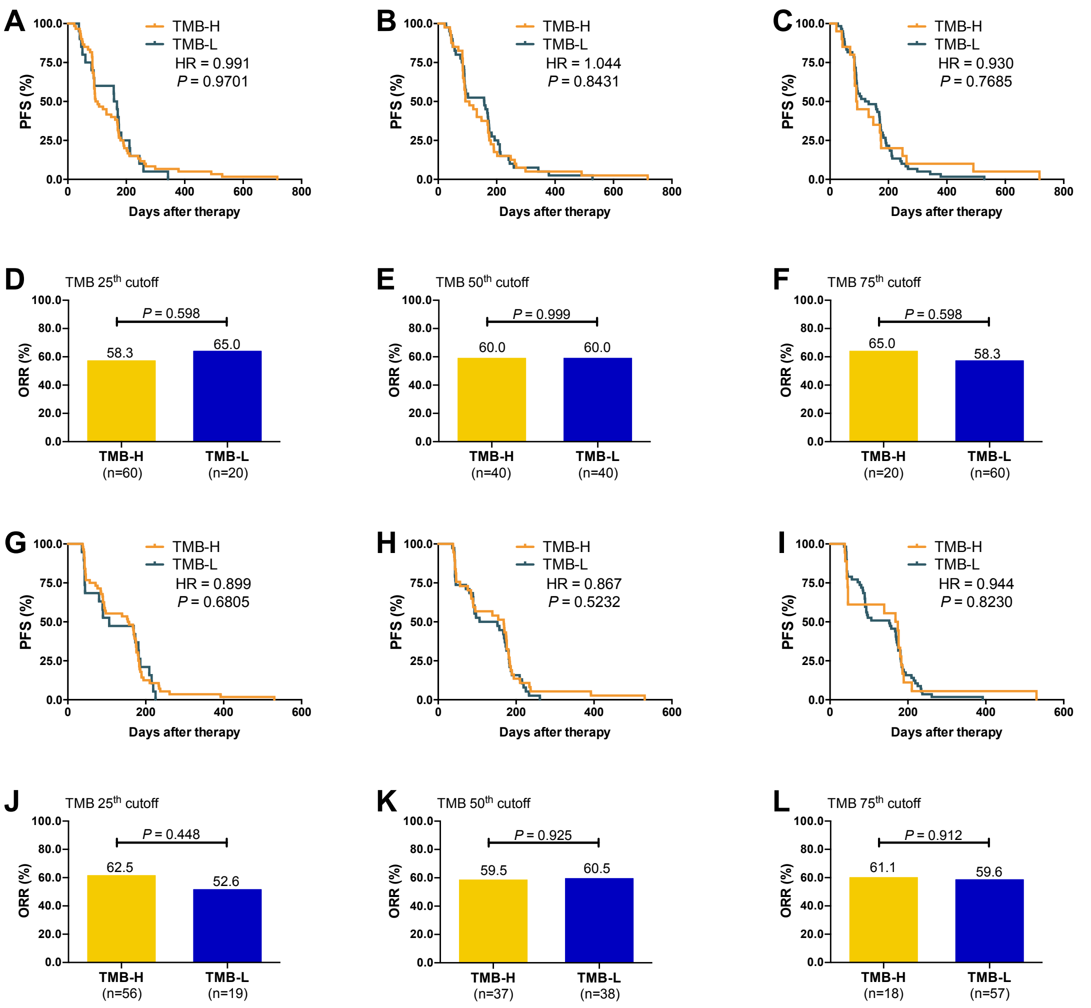

Supplemental Figure S7. Subgroup analysis of predictive value of TMB in LP and GP group. A-C. PFS comparison between TMB high and low in LP group (A. TMB cutoff 25th percentile; B. TMB cutoff 50th percentile; C. TMB cutoff 75th percentile); D-F. ORR comparison between TMB high and low in LP group (D. TMB cutoff 25th percentile; E. TMB cutoff 50th percentile; F. TMB cutoff 75th percentile); G-I. PFS comparison between TMB high and low in GP group (G. TMB cutoff 25th percentile; H. TMB cutoff 50th percentile; I. TMB cutoff 75th percentile); J-L. ORR comparison between TMB high and low in GP group (J. TMB cutoff 25th percentile; K. TMB cutoff 50th percentile; L. TMB cutoff 75th percentile). LP, paclitaxel liposome plus cisplatin; GP, gemcitabine plus cisplatin.

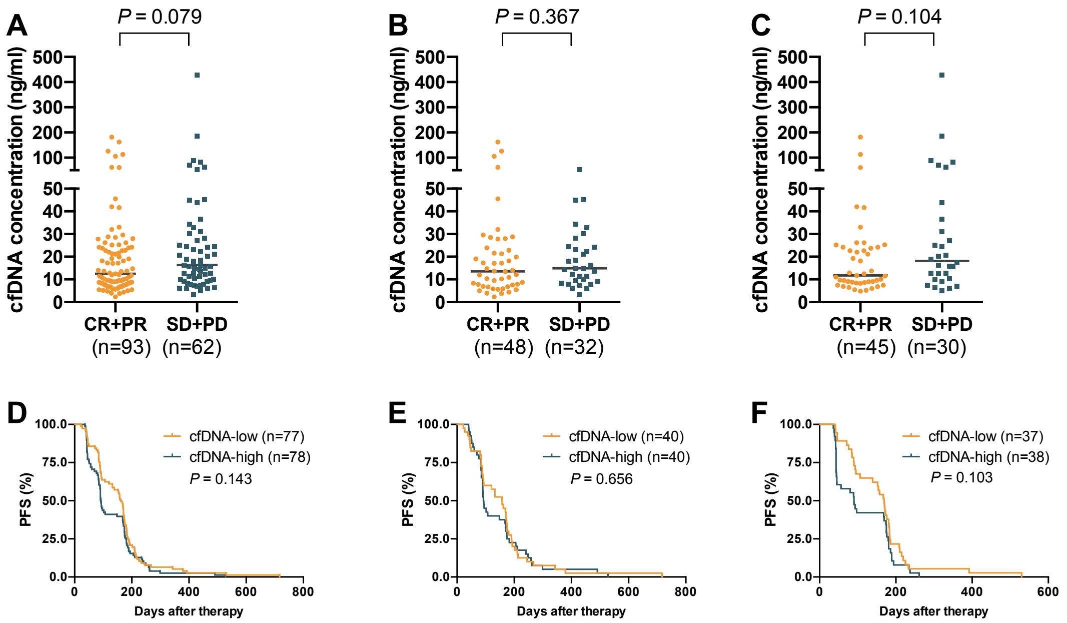

Supplemental Figure S8. Predictive value of cfDNA concentration at baseline. A. cfDNA concentration comparison between patients with CR+PR and SD+PD in all cases; B. cfDNA concentration comparison between patients with CR+PR and SD+PD in LP group; C. cfDNA concentration comparison between patients with CR+PR and SD+PD in GP group; D. PFS comparison between cfDNA concentration high and low group in all cases; E. PFS comparison between cfDNA concentration high and low group in LP group; F. PFS comparison between cfDNA concentration high and low group in GP group. LP, paclitaxel liposome plus cisplatin; GP, gemcitabine plus cisplatin.

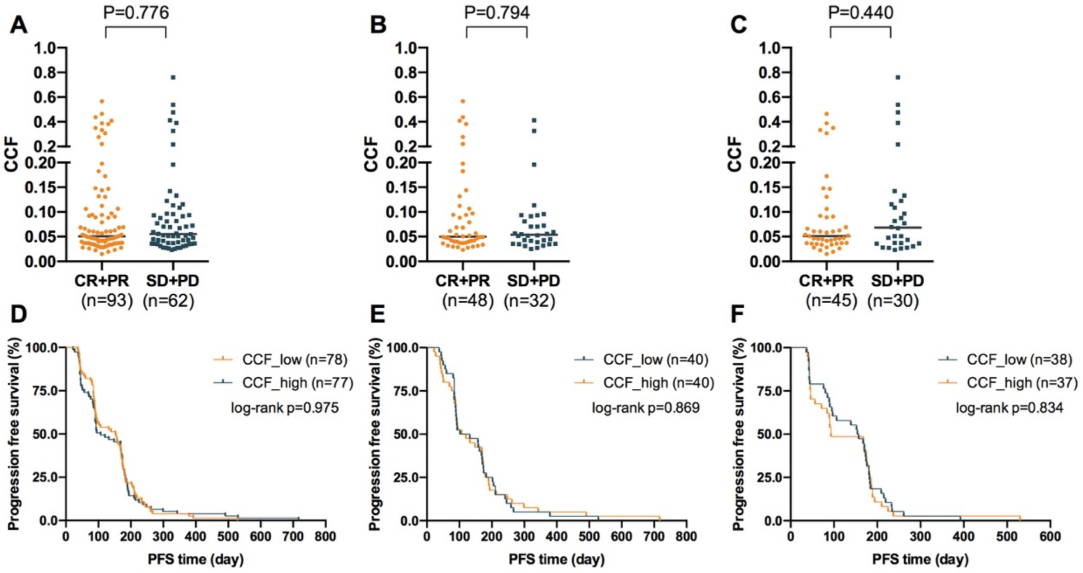

Supplemental Figure S9. Predictive value of fraction of circulating tumor DNA (ctDNA) at baseline. A. ctDNA fraction comparison between patients with CR+PR and SD+PD in all cases; B. ctDNA fraction comparison between patients with CR+PR and SD+PD in LP group; C. ctDNA fraction comparison between patients with CR+PR and SD+PD in GP group; D. PFS comparison between ctDNA fraction high and low group in all cases; E. PFS comparison between ctDNA fraction high and low group in LP group; F. PFS comparison between ctDNA fraction high and low group in GP group. LP, paclitaxel liposome plus cisplatin; GP, gemcitabine plus cisplatin; CCF, ctDNA fraction.

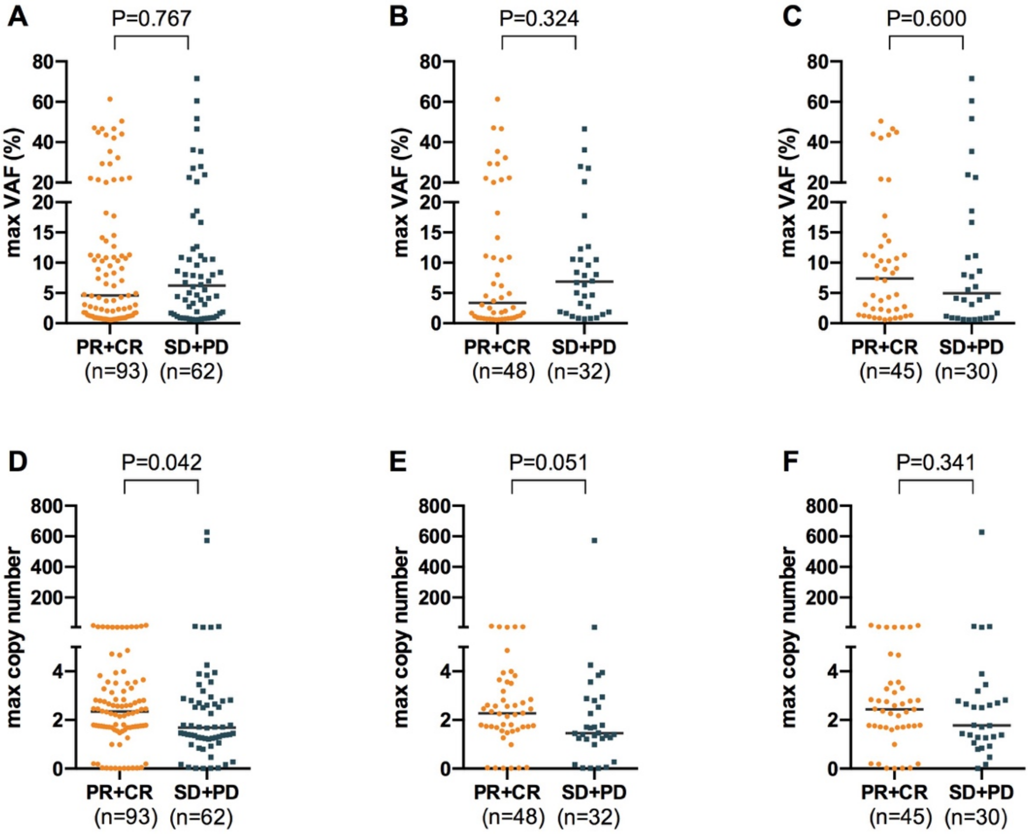

Supplemental Figure S10. Potential impact of maximum VAF of SNV and CNV on therapeutic response. A. maximum VAF of SNV comparison between patients with CR+PR and SD+PD in all cases; B. maximum VAF of SNV comparison between patients with CR+PR and SD+PD in LP group; C. maximum VAF of SNV comparison between patients with CR+PR and SD+PD in GP group; D. maximum CNV comparison between patients with CR+PR and SD+PD in all cases; E. maximum CNV comparison between patients with CR+PR and SD+PD in LP group; F. maximum CNV comparison between patients with CR+PR and SD+PD in GP group. Max, maximum; VAF, variant allele frequency.

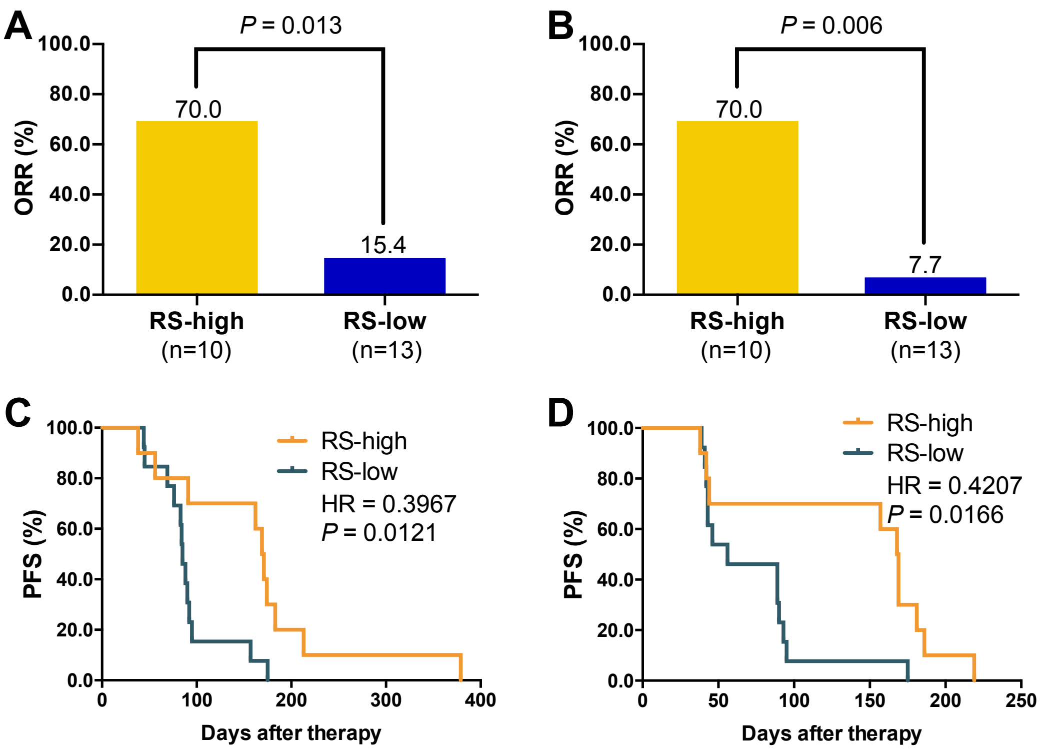

Supplemental Figure S11. Subgroup analysis of relationship between RS and treatment outcomes in training set. A. ORR comparison between RS high and low group in LP group; B. ORR comparison between RS high and low group in GP group; C. PFS comparison between RS high and low group in LP group; D. PFS comparison between RS high and low group in GP group. LP, paclitaxel liposome plus cisplatin; GP, gemcitabine plus cisplatin.

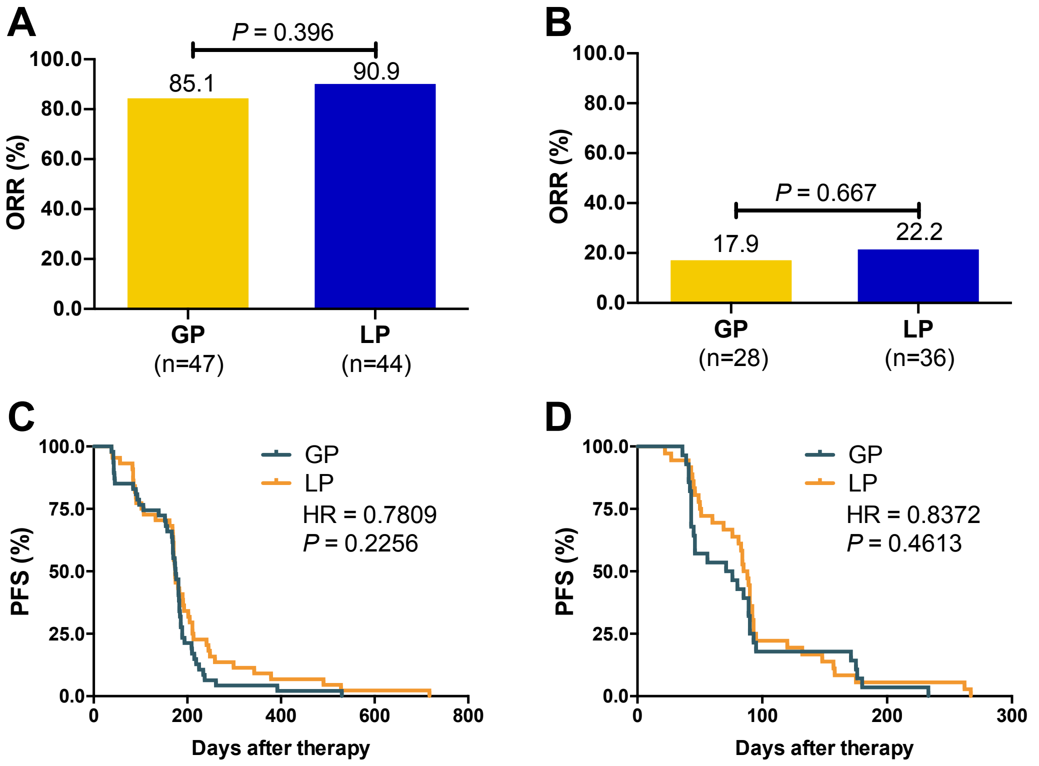

Supplemental Figure S12. Distinguishable value of RS in patients received different chemotherapeutic regimen in training set plus validation set. A. ORR comparison between LP and GP group in RS high group; B. ORR comparison between LP and GP group in RS low group; C. PFS comparison between LP and GP group in RS high group; D. PFS comparison between LP and GP group in RS low group. LP, paclitaxel liposome plus cisplatin; GP, gemcitabine plus cisplatin.

A

	Total	RS high	%	RS low	%	*P* value
Age						
< 65	27	16	72.7%	11	55.0%	0.231
> 65	15	6	27.3%	9	45.0%	
Gender						
Male	30	15	68.2%	15	75.0%	0.625
Female	12	7	31.8%	5	25.0%	
ECOG PS						
0	13	8	36.4%	5	25.0%	0.426
1	29	14	63.6%	15	75.0%	
Histology						
Squamous	18	11	50.0%	7	35.0%	0.327
Non-squamous	24	11	50.0%	13	65.0%	
Regimen						
Docetaxel + cisplatin	29	14	63.6%	15	75.0%	0.426
Docetaxel + carboplatin	13	8	36.4%	5	25.0%	
Response rate						
Complete response	0	0	0.0%	0	0.0%	
Partial response	15	12	54.5%	3	15.0%	0.019
Stable disease	19	8	36.4%	11	55.0%	
Progression disease	8	2	9.1%	6	30.0%	

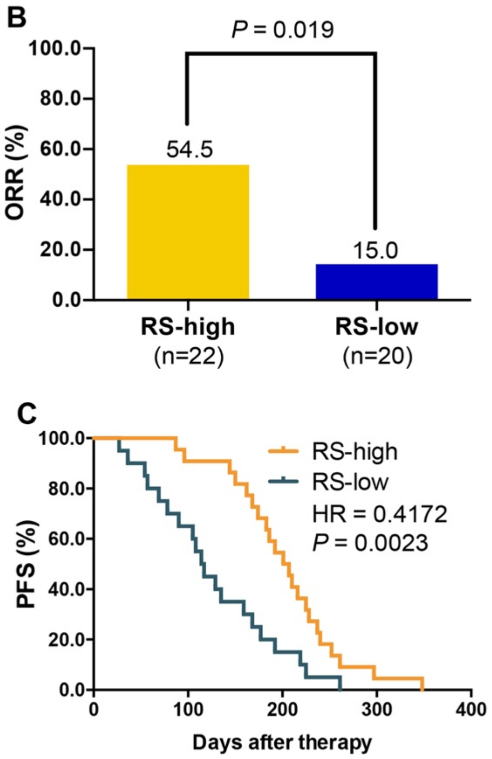

Supplemental Figure S13. Predictive value of RS in a real-world cohort. A. baseline features of 42 patients with NSCLC received first-line chemotherapy; B. ORR comparison between RS high and low group; C. PFS comparison between RS high and low group.

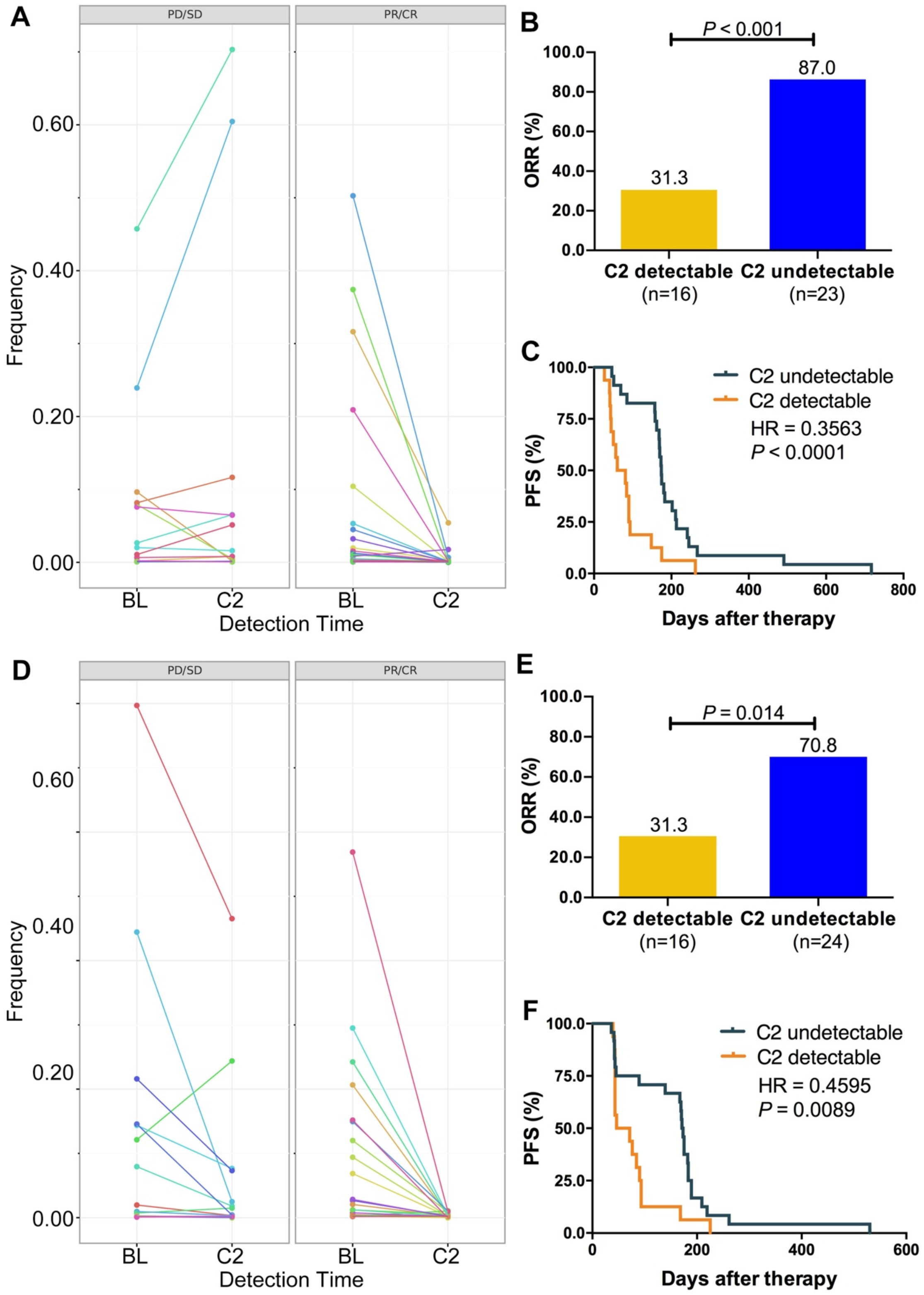

Supplemental Figure S14. Subgroup analysis of ICP-based dynamic change of VAF monitored the treatment response. A. ICP-based change of VAF between CR/PR and SD/PD at baseline and after 2 cycles treatment in LP group; B. ORR comparison between VAF detected and not detected after

2 cycles treatment in LP group; C. Kaplan-Meier curve of PFS comparison between VAF detected and not detected after 2 cycles treatment in LP group; D. ICP-based change of VAF between CR/PR and SD/PD at baseline and after 2 cycles treatment in GP group; E. ORR comparison between VAF detected and not detected after 2 cycles treatment in GP group; F. Kaplan-Meier curve of PFS comparison between VAF detected and not detected after 2 cycles treatment in GP group. LP, paclitaxel liposome plus cisplatin; GP, gemcitabine plus cisplatin.

Supplemental Table S1. Baseline characteristics of included patients (n = 155).

		Total		LP (n=80)		GP (n=75)		*P* value
Age	< 65	104	67.1%	48	60.0%	56	74.7%	0.052
	≥ 65	51	32.9%	32	40.0%	19	25.3%	
Gender	Male	151	97.4%	77	96.3%	74	98.7%	0.659
	Female	4	2.6%	3	3.8%	1	1.3%	
ECOG PS	0	26	16.8%	14	17.5%	12	16.0%	0.803
	1	129	83.2%	66	82.5%	63	84.0%	
Smoking history	Never	6	3.9%	3	3.8%	3	4.0%	0.737
	Ever/current	149	96.1%	77	96.3%	72	96.0%	
Response rate	CR	1	0.6%	1	1.3%	0	0.0%	0.999
	PR	92	59.4%	47	58.8%	45	60.0%	
	SD	28	18.1%	18	22.5%	10	13.3%	
	PD	34	21.9%	14	17.5%	20	26.7%	

ECOG PS: Eastern Cooperative Oncology Group performance status; LP: paclitaxel liposome plus cisplatin; GP: gemcitabine plus cisplatin; TMB: tumor mutational burden; CR: complete response; PR: partial response; SD: stable disease; PD: progression disease.

Supplemental Table S2. Panel 1 gene list.

ABCA13	ATR	CCND1	CRLF2	ERBB3	FMO1	HSPA1B	KPNA4	MSH2	NTRK1
ABCA8	ATRX	CCND2	CSF1R	ERBB4	FOLH1	HSPA4	KPNB1	MSH3	NTRK2
ABCB1	AURKA	CCND3	CSF3R	ERCC1	FOXL2	HSPA5	KRAS	MSH6	NTRK3
ABCC2	AURKB	CCNE1	CTCF	ERCC2	FOXP1	HYOU1	LAMA3	MTF1	NUP85
ABL1	AXIN1	CCR4	CTNNB1	ERCC4	FRAS1	IARS	LEPR	MTHFR	NUP93
ACADSB	AXL	CD274	CUL3	ERG	FUBP1	ID2	LMO1	MTOR	OTOS
ACOT13	B2M	CD40	CXCL8	ERI1	FUS	ID3	LONRF3	MTR	P2RY8
ADAMTS6	BAP1	CD74	CXCR4	ERRFI1	GABRP	IDH1	LRP2	MTRR	PALB2
ADRB1	BARD1	CD79A	CYBA	ESR1	GALNT14	IDH2	LRRC34	MUTYH	PAPOLG
ADSS	BCL2	CD79B	CYFIP1	ETV1	GANC	IGF1R	LYN	MYADM	PAQR8
AK7	BCL2L1	CDA	CYLD	ETV6	GATA1	IGF2	MAGOHB	MYC	PARP1
AKT1	BCOR	CDC25B	CYP19A1	EWSR1	GATA2	IKBKE	MALT1	MYCL	PAX5
AKT2	BCYRN1	CDC73	CYP2B6	EXOSC8	GATA3	IKZF1	MAP2K1	MYCN	PBRM1
AKT3	BLM	CDH1	CYP2C19	EZH2	GLI1	IL7R	MAP2K2	MYD88	PDCD1
ALG9	BRAF	CDK12	CYP2C8	EZR	GMEB1	INHBA	MAP2K4	MYO10	PDCD1LG2
ALK	BRCA1	CDK4	CYP2D6	F13A1	GNA11	INPP4B	MAP3K1	NAB1	PDE6C
ALOX12B	BRCA2	CDK6	DAXX	FAM149A	GNA13	IPO7	MAP3K4	NAB2	PDGFB
AMER1	BRD4	CDK7	DBT	FAM153B	GNAQ	IRAK1	MAP4K5	NBN	PDGFRA
ANKRA2	BRIP1	CDK8	DDR2	FANCA	GNAS	IRF4	MAPK1	NCOA6	PDGFRB
ANKRD46	BRS3	CDKL3	DEPDC5	FANCC	GPAT3	IRF6	MAPKAP1	NDUFS1	PDPN
ANO1	BTF3	CDKN1A	DHFR	FANCD2	GPM6A	IRF8	MAPKBP1	NEO1	PGBD1
APC	BTG1	CDKN1B	DIAPH1	FANCG	GRIN2A	IRS2	MARK2	NF1	PIGF
APOL2	BTK	CDKN2A	DICER1	FANCI	GSK3B	ITGAL	MCL1	NF2	PIK3C2G
APOPT1	C20orf96	CDKN2B	DIS3	FAS	GSTA1	JAK1	MDM2	NFE2L2	PIK3CA
AR	C22orf23	CDKN2C	DNMT3A	FAT1	GSTM1	JAK2	MDM4	NFKBIA	PIK3CB

ARAF	C2CD6	CDO1	DOCK11	FBXW7	GSTP1	JAK3	MED12	NFXL1	PIK3CG
ARHGAP4	C5orf15	CEBPA	DOT1L	FGF16	H3F3A	JUN	MED19	NKAP	PIK3R1
ARHGAP6	C8orf34	CEP120	DPYD	FGF19	HAUS2	KCNJ2	MEF2B	NKX2-1	PIK3R2
ARID1A	C9orf72	CEP290	DROSHA	FGF3	HAUS6	KDM5A	MEIS1	NLRP7	PLCG2
ARID1B	CAB39	CHD1	DSCAM	FGF4	HCAR2	KDM5C	MEN1	NOTCH1	PLEKHA1
ARID2	CALD1	CHEK1	DYNC2H1	FGFR1	HEY1	KDM6A	MET	NOTCH2	PLEKHH2
ARID4A	CALM2	CHEK2	EGFR	FGFR2	HGF	KDR	MIA2	NOTCH3	PMS2
ARL6IP6	CALR	CIC	EIF4G3	FGFR3	HLA-A	KEAP1	MITF	NPM1	PNO1
ARMC5	CARD11	CNKSR3	EML4	FGFR4	HLA-B	KIAA1210	MLH1	NR1I3	POLD1
ARPC2	CASP8	CNOT8	EP300	FH	HLA-C	KIF5B	MMP16	NR4A3	POLE
ASH1L	CAST	COL15A1	EPHA3	FLCN	HLA-DRB1	KIR3DX1	MMP3	NRAS	PPARG
ASXL1	CBFB	COX18	EPHA5	FLOT1	HNF1A	KIT	MOV10L1	NSD1	PPHLN1
ATIC	CBL	CPLANE1	EPHA7	FLT1	HNF4A	KMT2A	MPL	NSD2	PPP2R1A
ATM	CBR3	CREBBP	EPHB1	FLT3	HNRNPH1	KMT2C	MRE11	NSD3	PRDM1
ATP9B	CBR4	CRKL	ERBB2	FLT4	HRAS	KMT2D	MRPL19	NT5C2	PREX2
PRKAR1A	RAD51C	RIPK2	SEL1L3	SLIT1	SRC	TBC1D8B	TOE1	UBE3C	ZDHHC17
PRKCI	RAD51D	RNF19A	SEMA3C	SMAD2	SS18	TBX3	TOP1	UGT1A1	ZMYM4
PRKN	RAD52	RNF43	SETD2	SMAD3	STAG2	TECPR2	TOP2B	ULK4	ZNF2
PRPF39	RAD54L	ROS1	SF3B1	SMAD4	STARD4	TENT5C	TP53	UMPS	ZNF367
PTCH1	RAF1	RPA4	SFXN4	SMARCA4	STAT3	TERT	TPH1	UPF2	ZNF711
PTEN	RARA	RPTOR	SHROOM3	SMARCB1	STK11	TET2	TRA2A	VEGFA	ZNF805
PTPN11	RB1	RRM1	SIMC1	SMO	STMN1	TGFBR2	TRIM24	VHL	ZNF91
PTPRJ	RBM10	RRP1B	SIPA1L2	SNX6	STRBP	TMEM67	TSC1	VSIG10	ZZZ3
PURA	RBM27	RUNX1	SLC22A2	SOCS1	STYX	TMPRSS15	TSC2	WDR5	
RABGAP1L	REL	RYR2	SLC30A5	SOD2	SUCLG1	TMPRSS2	TSHR	WT1	
RAC1	RET	SASH1	SLC31A1	SOX2	SUFU	TNFAIP3	TSN	WWC3	

RAD21	RFC1	SDHA	SLC34A2	SOX9	SUGCT	TNFRSF14	TXNRD1	XPC
RAD50	RHOT1	SDHB	SLC7A8	SPC24	SYK	TNFSF13B	TYMS	XPO1
RAD51	RIC1	SDHC	SLCO1B1	SPEN	TAF15	TNKS	U2AF1	XRCC1
RAD51B	RICTOR	SDHD	SLCO1B3	SPOP	TAGAP	TNRC18	UBE2E3	ZBBX

Supplemental Table S3. Panel 2 gene list.

AKT1	DDR2	FGFR1	MAP2K1	PDGFRA	SMAD4
ALK	EGFR	FGFR2	MET	PIK3CA	STK11
APC	ERBB2	FGFR3	NOTCH1	PTEN	TP53
BRAF	ERBB4	KIT	NRAS	RET	UGT1A1
CTNNB1	FBXW7	KRAS	NTRK1	ROS1	

References

1. DePristo MA, Banks E, Poplin R, et al. A framework for variation discovery and genotyping using next-generation DNA sequencing data. *Nat Genet* 2011;43:491-8.

2. Lai Z, Markovets A, Ahdesmaki M, et al. VarDict: a novel and versatile variant caller for next-generation sequencing in cancer research. *Nucleic Acids Res* 2016;44:e108.

3. Wang K, Li M and Hakonarson H. ANNOVAR: functional annotation of genetic variants from high-throughput sequencing data. *Nucleic Acids Res* 2010;38:e164.

4. Karczewski KJ, Weisburd B, Thomas B, et al. The ExAC browser: displaying reference data information from over 60 000 exomes. *Nucleic Acids Res* 2017;45:D840-D845.

5. Han T, Yu J, Lin X, et al. A new method towards calculating the cancer cell fraction in cell-free DNA. *Journal of Clinical Oncology* 2019;37:e13053-e13053.

6. Chang MT, Asthana S, Gao SP, et al. Identifying recurrent mutations in cancer reveals widespread lineage diversity and mutational specificity. *Nat Biotechnol* 2016;34:155-63.

7. Chang MT, Bhattarai TS, Schram AM, et al. Accelerating Discovery of Functional Mutant Alleles in Cancer. *Cancer Discov* 2018;8:174-183.

8. Chen N. Using RepeatMasker to identify repetitive elements in genomic sequences. *Curr Protoc Bioinformatics* 2004;Chapter 4:Unit 4 10.

9. Consortium EP. The ENCODE (ENCyclopedia Of DNA Elements) Project. *Science* 2004;306:636-40.
